# Lactate preconditioning promotes a HIF-1α-mediated metabolic shift from OXPHOS to glycolysis in normal human diploid fibroblasts

**DOI:** 10.1038/s41598-020-65193-9

**Published:** 2020-05-20

**Authors:** Alexandra M. Kozlov, Asad Lone, Dean H. Betts, Robert C. Cumming

**Affiliations:** 1 Department of Biology, The University of Western Ontario, London, Ontario N6A 5B7 Canada; 2 Department of Physiology and Pharmacology, Schulich School of Medicine and Density, The University of Western Ontario, London, Ontario N6A 5C1 Canada; 3 Department of Obstetrics and Gynaecology, Schulich School of Medicine and Dentistry, The University of Western Ontario, London, Ontario N6A 5W9 Canada

**Keywords:** Reprogramming, Transcription, Cell biology, Molecular biology

## Abstract

Recent evidence has emerged that cancer cells can use various metabolites as fuel sources. Restricting cultured cancer cells to sole metabolite fuel sources can promote metabolic changes leading to enhanced glycolysis or mitochondrial OXPHOS. However, the effect of metabolite-restriction on non-transformed cells remains largely unexplored. Here we examined the effect of restricting media fuel sources, including glucose, pyruvate or lactate, on the metabolic state of cultured human dermal fibroblasts. Fibroblasts cultured in lactate-only medium exhibited reduced PDH phosphorylation, indicative of OXPHOS, and a concurrent elevation of ROS. Lactate exposure primed fibroblasts to switch to glycolysis by increasing transcript abundance of genes encoding glycolytic enzymes and, upon exposure to glucose, increasing glycolytic enzyme levels. Furthermore, lactate treatment stabilized HIF-1α, a master regulator of glycolysis, in a manner attenuated by antioxidant exposure. Our findings indicate that lactate preconditioning primes fibroblasts to switch from OXPHOS to glycolysis metabolism, in part, through ROS-mediated HIF-1α stabilization. Interestingly, we found that lactate preconditioning results in increased transcript abundance of *MYC* and *SNAI1*, key facilitators of early somatic cell reprogramming. Defined metabolite treatment may represent a novel approach to increasing somatic cell reprogramming efficiency by amplifying a critical metabolic switch that occurs during iPSC generation.

## Introduction

The preferential use of glycolysis even in the presence of oxygen is known as aerobic glycolysis or the Warburg effect, a unique form of metabolism originally identified in cancer cells, but also found in many non-transformed cells^1,2^. By shuttling glucose primarily through glycolysis, cancer cells fuel their proliferation by accumulating glycolytic intermediates required for fatty acid and nucleic acid synthesis. As lactate is a by-product of glycolysis, cancer cells exist in an acidic microenvironment which serves to facilitate angiogenesis and tumour invasion^3^. Research examining the relationship between cancer cells and their microenvironment has revealed a novel phenomenon known as the reverse Warburg effect^4^. The reverse Warburg effect is based on the theory that cells within a tumour can switch between glycolysis and oxidative phosphorylation (OXPHOS)^5–7^. The reverse Warburg effect postulates that oxidative cancer cells secrete reactive oxygen species (ROS) which induce oxidative stress in surrounding stromal cells such as cancer-associated fibroblast (CAF) cells^8,9^. To respond to this stress, CAFs adopt aerobic glycolysis as their primary form of metabolism^8,9^. Glycolytic CAFs secrete lactate and precursors for nucleic acid and fatty acid synthesis, which are then taken up by adjacent cancer cells to fuel OXPHOS and support proliferation, thereby continuing the cycle^8,9^.

Long considered merely a by-product of glycolysis, lactate is emerging as an important signalling molecule and energy source^10–15^. In addition to promoting angiogenesis and tumour invasion, lactate has been shown to promote ROS production through the Fenton reaction and prevent the degradation of the transcription factor, hypoxia inducible factor one alpha (HIF-1α)^15,16^. With respect to lactate as a fuel source, extracellular lactate can be taken up by monocarboxylic acid transporters (MCTs) and, in a reaction catalysed by lactate dehydrogenase B (LDHB), undergo conversion to pyruvate for subsequent entry into the tricarboxylic acid (TCA) cycle^13^. Various cancer cell types preferentially uptake lactate to fuel adenosine triphosphate (ATP) production, even in the presence of sufficient glucose^17^. Indeed, oxygenated cancer cells can utilize glucose, lactate and L-glutamine in concert to optimize ATP production while supporting their biosynthetic needs^10^. Furthermore, studies have demonstrated that restricting cancer cells to a single fuel source such as pyruvate, can induce a metabolic switch from glycolysis to OXPHOS, further demonstrating the metabolic plasticity of cancer cells^18^.

Many non-cancerous cells exhibit phenotypic changes that are dependent on metabolic switching between glycolysis and OXPHOS. For example, both embryonic and adult stem cells exhibit high metabolic plasticity, and switch from glycolysis to OXPHOS during differentiation^19^. In contrast, the generation of induced pluripotent stem cells (iPSCs) from human somatic cells is dependent on shifting cellular metabolism from OXPHOS to glycolysis^19^. Due to their developmental potential, patient-derived iPSCs represent a powerful tool that can be used for personalized disease modelling, drug discovery and cell replacement therapy^20^. Unfortunately, the process of somatic cell reprogramming is vastly inefficient^21^. Moreover, the ectopic expression of transcription factors used to drive reprogramming raises safety concerns^21,22^. The original cocktail of transcription factors used to generate iPSCs from somatic cells are referred to as Yamanaka factors (OCT4, SOX2, KLF4, c-MYC: OSKM)^23,24^. OSK are the core factors responsible for acquisition of pluripotent stem cell identity^25^. Enhancer factors such as NANOG, GLIS1, SALL4, c-MYC, and LIN28 have been used in combination with OSK or OS to increase reprogramming efficiency^25,26^. While *NANOG, GLIS1, SALL4,* and *LIN28* are normally expressed during early embryonic development, *MYC* is a protooncogene which promotes early phase metabolic remodelling, and ultimately increased glycolysis, during reprogramming^27^.

Initial studies demonstrated that enhancing glycolytic metabolism through exposure to hypoxic culture conditions increases reprogramming efficiency^28,29^. Furthermore, several studies show that c-MYC can be replaced by exposure to pharmacological activators of pyruvate dehydrogenase kinase 1 (PDK1), an enzyme involved in promoting glycolytic metabolism^30^. However, the role of OXPHOS during somatic cell reprogramming is less defined. ROS are natural by-products of mitochondrial electron transport chain (ETC) activity^31^. Although ROS are important signalling molecules implicated in cell stress response and development, excess ROS is cytotoxic^32^. Following Yamanaka factor transduction, somatic cells become increasingly oxidative, culminating in a burst of ROS production; events shown to be necessary for promoting a “metabolic switch” to glycolytic metabolism and subsequent pluripotency acquisition^33^. Indeed, hindering OXPHOS and/or ROS production before the metabolic switch blunts reprogramming efficiency^34–36^. Thus, modulation of metabolic flux between glycolysis and OXPHOS presents a unique opportunity to either enhance reprogramming efficiency or reduce the number of Yamanaka factors required for iPSC generation.

While the effects of metabolite fuel restriction on cancer cell metabolism has been examined, the effects of metabolite restriction on non-transformed cells is less understood. We postulated that culturing normal diploid human fibroblast cells in culture medium containing a defined metabolite fuel source could be used to trigger a metabolic switch from OXPHOS to glycolysis or vice versa. In this study we examined the metabolic effects of restricting human foreskin dermal fibroblast cells to medium containing only glucose, pyruvate or lactate as a fuel source. We found that preconditioning fibroblasts to culture medium containing only lactate as a fuel source results in a switch from OXPHOS to glycolysis, in part, through ROS-mediated stabilization of HIF-1α. Furthermore, this preconditioning strategy resulted in increased transcript abundance of early somatic cell reprogramming regulators *MYC* and *SNAI1*. Using this novel strategy to force a metabolic switch may provide a simple method of increasing somatic cell reprogramming efficiency. In addition, lactate preconditioning could also provide an alternative replacement for c-MYC, thereby providing a safer strategy for iPSC generation and translational applications.

## Results

### Defined metabolite treatment alters the metabolism of normal human fibroblast cells

**Figure 1:**
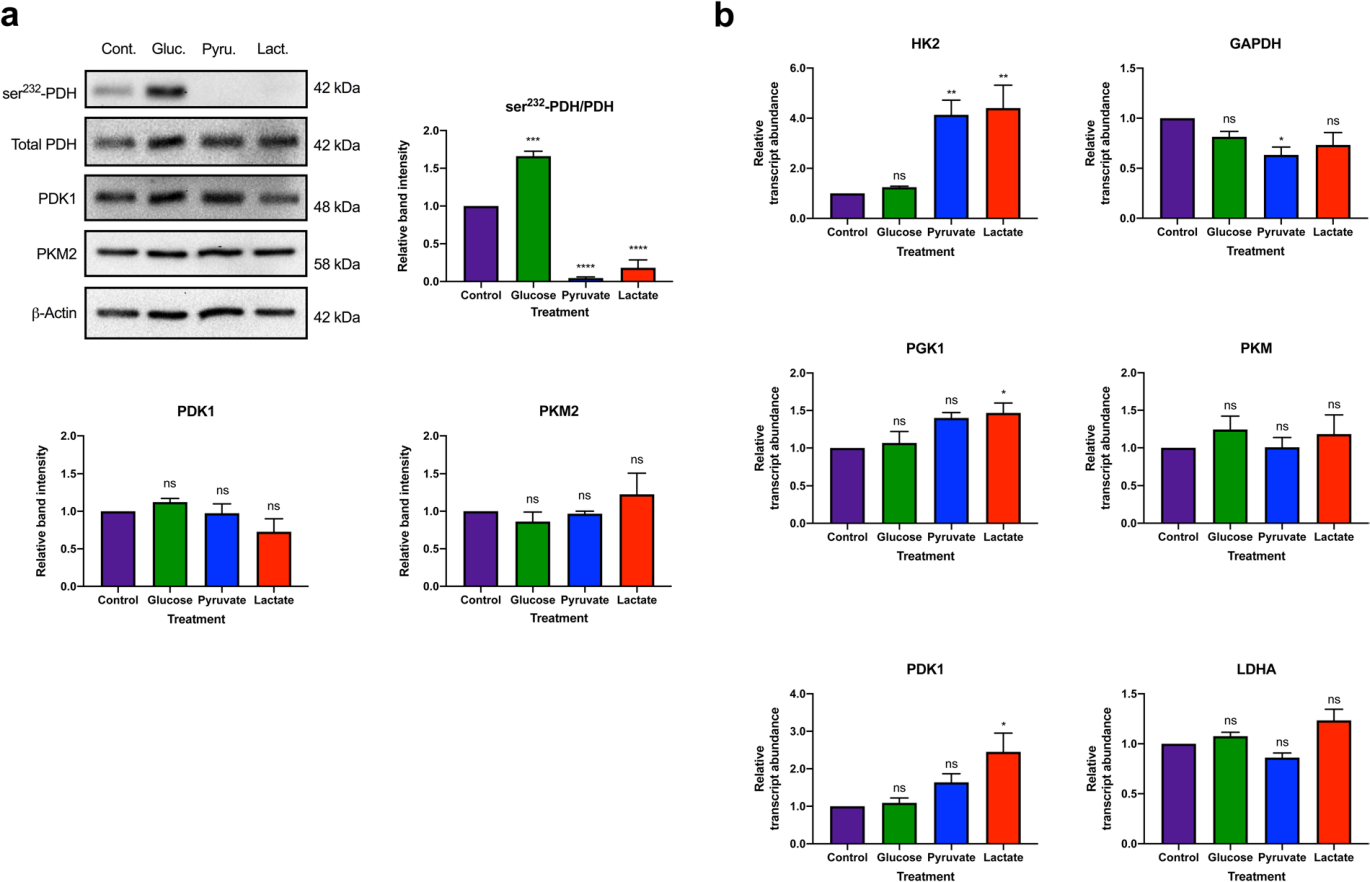
Defined metabolite treatment promotes post translational and transcriptional changes in human fibroblasts. BJ fibroblasts were cultured in defined metabolite media for 24 h prior to protein harvest and RNA isolation. (**a**) Immunoblots were probed with antibodies directed against the indicated metabolic markers for glycolysis and OXPHOS. Densitometric analysis of the ratio of ser^232^-PDH to total PDH band intensities normalized to β-Actin, revealed that BJ cells treated with glucose promoted significantly increased phosphorylation of PDH (indicative of glycolysis), whereas treatment with pyruvate or lactate resulted in significantly decreased phosphorylation of PDH (indicative of OXPHOS) compared to control-treated cells. Densitometric analysis of PKM2 and PDK1 band intensities normalized to β-Actin, revealed that 24 h defined metabolite treatment did not alter PKM2 or PDK1 protein abundance in BJ cells compared to control conditions. (**b**) qRT-PCR using *ACTB* and *RPL37A* as housekeeping genes, revealed that lactate-treatment significantly increased transcription of genes encoding the glycolytic enzymes, HK2, PGK1 and PDK1 compared to control. Pyruvate treatment resulted in a significant increase and decrease in the transcript abundance of genes enocding HK2 and GADPH, respectively, compared to control. The data presented represent N = 3 ± s.e.m. All qRT-PCR was performed in triplicate. The immunoblots are representative of three independent experiments. Full length blots can be found in Supplementary Fig. S4. Asterisks indicate significant difference (p < 0.05 = *, p < 0.01 = **, p < 0.001 = ***, p < 0.0001 = ****) and ns = no difference tested by One-way ANOVA and Dunnett’s multiple comparisons test.

To determine if normal cells were capable of using alternative fuel sources in the absence of glucose, we investigated the impact of restricted fuel source availability on human foreskin dermal fibroblast (BJ) cell metabolism. To this end, BJ fibroblasts were initially cultured in medium containing 20 mM glucose, pyruvate, or lactate as the sole metabolite fuel source for 24 h. We first examined the impact of metabolite restriction on the protein and transcript abundance of metabolic enzymes. Pyruvate dehydrogenase (PDH) catalyses the conversion of pyruvate to acetyl-CoA, ultimately facilitating ATP production by OXPHOS^37^. Phosphorylation of PDH by PDK1 results in inhibition of PDH activity and renders cells more dependent on glycolysis to meet their energy needs^38^. Pyruvate kinase is the enzyme responsible for catalysing the conversion of phosphoenolpyruvate (PEP) to pyruvate, the last step in glycolysis^38,39^. Pyruvate kinase muscle isozyme 2 (PKM2) is an alternatively spliced isozyme of pyruvate kinase that, following phosphorylation, can translocate to the nucleus and facilitate increased transcription of enzymes that favour lactate production and glycolysis^40^. Immunoblot analysis revealed that BJ cells restricted to medium containing only glucose as a fuel source exhibited a significantly increased ratio of ser^232^-PDH to total PDH (p < 0.001) compared to cells cultured under control conditions. In contrast, BJ cells restricted to pyruvate or lactate as a fuel source exhibited a significantly decreased ratio of ser^232^-PDH to total PDH (p < 0.0001) compared to control (Fig. 1a). However, none of the metabolite-restricted media altered PKM2 or PDK1 protein levels (Fig. 1a). Interestingly, both pyruvate- and lactate-treated fibroblast cells exhibited significantly increased transcript abundance of *hexokinase 2* (*HK2*) (p < 0.01), which enocodes the protein that catalyses the first step in glycolysis, compared to control (Fig. 1b)^39^. Furthermore, only lactate-treated BJ fibroblasts exhibited significantly increased transcript abundance of *PDK1* (p < 0.05) and *phosphoglycerate kinase 1* (*PGK1*) (p < 0.05), with a small but non-significant increase in *lactate dehydrogenase A* (*LDHA*) and *PKM* transcript abundance compared to control (Fig. 1b). In contrast, pyruvate-treated BJ fibroblasts exhibited significantly decreased *glyceraldehyde 3-phosphate dehydrogenase* (*GADPH*) (p < 0.05) transcript abundance compared to control (Fig. 1b). While GAPDH and PGK1 catalyse the sixth and seventh step of glycolysis, respectively, LDHA converts pyruvate to lactate at the end of glycolysis^39,41^. Defined metabolite treatment had no effect on the transcript abundance of tricarboxylic acid (TCA) cycle genes, *ATP citrate lyase* (*ACLY*), *isocitrate dehydrogenase 1* (*IDH1*), *oxoglutarate dehydrogenase* (*OGDH*), *malate dehydrogenase* (*MDH1*), and *succinate dehydrogenase complex iron sulphur subunit B* (*SDHB*) (*see* Supplementary Fig. S1). These initial findings suggest defined metabolite treatment primarily impacts glycolytic enzymes rather than OXPHOS.

**Figure 2:**
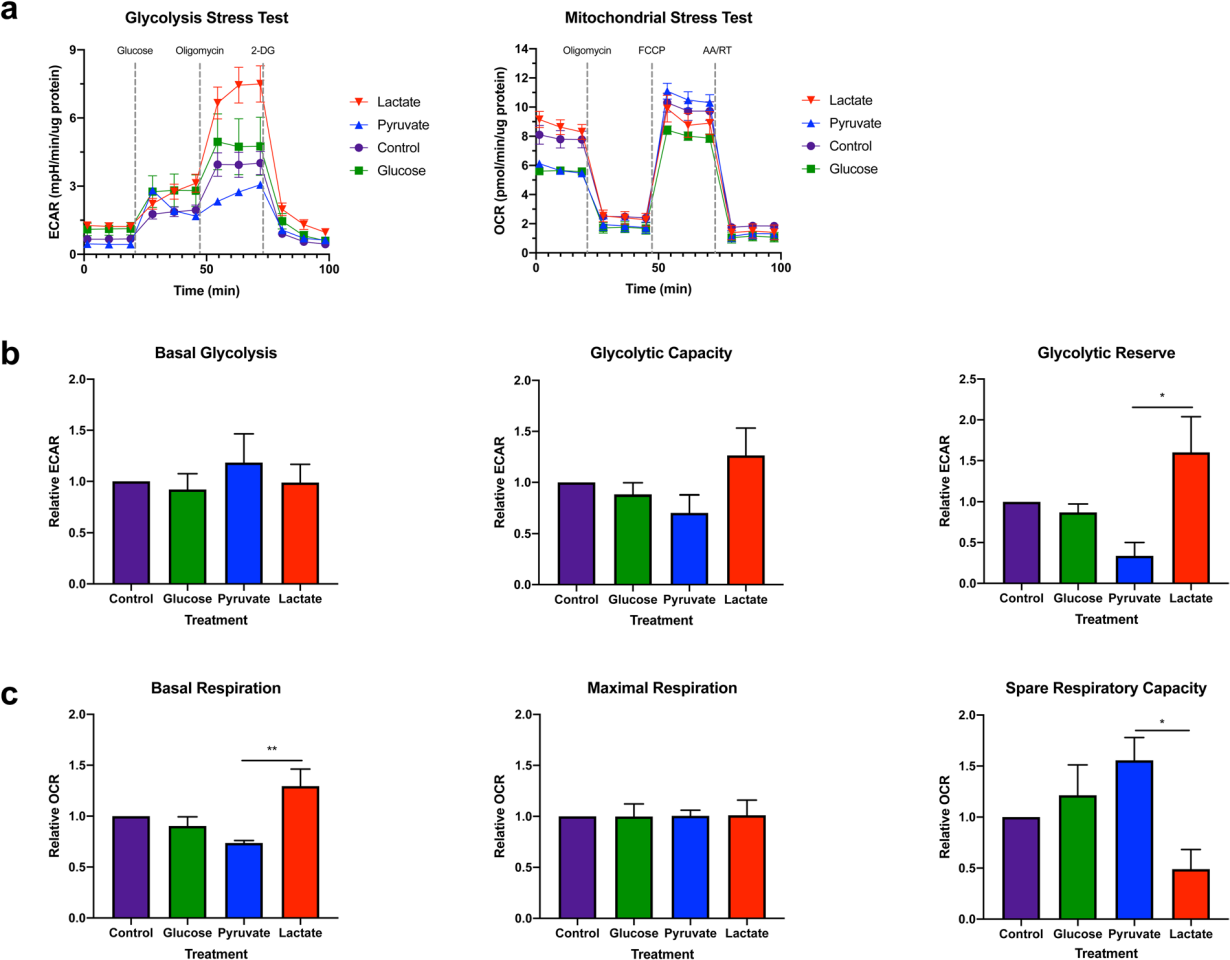
Lactate treatment promotes bivalent metabolism in fibroblasts. BJ fibroblast cells were cultured in defined metabolite media for 24 h prior to analysis with the Seahorse XF^e^24 Flux Analyzer. (**a**) Extracellular acidification rate (ECAR) normalized to total protein was used as proxy measure of glycolytic activity following subsequent injections of glucose, oligomycin and 2-deoxy-D-glucose (2-DG) during the glycolysis stress test. Oxygen consumption rate (OCR) normalized to total protein was used as a proxy measure of OXPHOS following subsequent injections of oligomycin, carbonyl cyanide-p-trifluoromethoxyphenylhydrazone (FCCP) and antimycin A/rotenone (AA/RT) during the mitochondrial stress test. (**b**) No difference in basal glycolysis or glycolytic capacity was observed following glucose and oligomycin injection, respectively. However, lactate-treated BJ cells exhibited a significantly greater glycolytic reserve than pyruvate-treated cells. (**c**) Basal respiration was significantly elevated in lactate-treated BJ fibroblast cells compared to pyruvate-treated cells. However, lactate-treated BJ cells exhibited significantly lower spare respiratory capacity than pyruvate-treated cells. Maximal respiration did not differ between treatments. The data presented represent N = 4 ± s.e.m. with 5 technical replicates per treatment. Asterisks indicate significant difference (p < 0.05 = *, p < 0.01 = **) and ns = no difference tested by One-way ANOVA and Tukey’s multiple comparisons test.

To validate the real time effect of defined metabolite treatment on BJ cell metabolism, extracellular acidification rate (ECAR) and oxygen consumption rate (OCR) were measured by the glycolysis stress test and the mitochondrial stress test respectively (Fig. 2a). Cells treated with different metabolites exhibited similar basal glycolysis, glycolytic capacity and maximal respiration (Fig. 2b,c). However, lactate-treated BJ cells exhibited a significantly greater glycolytic reserve compared to pyruvate-treated cells (p < 0.05) (Fig. 2b). While lactate-treated BJ cells also exhibited significantly greater basal respiration (p < 0.01) than pyruvate-treated cells, pyruvate-treated BJ fibroblasts exhibited a significantly greater spare respiratory capacity than lactate-treated cells (p < 0.05) (Fig. 2c). These results suggest that lactate-treated BJ fibroblasts exhibit a bivalent metabolism based on their ability to switch to glycolysis when glucose becomes available.

**Figure 3:**
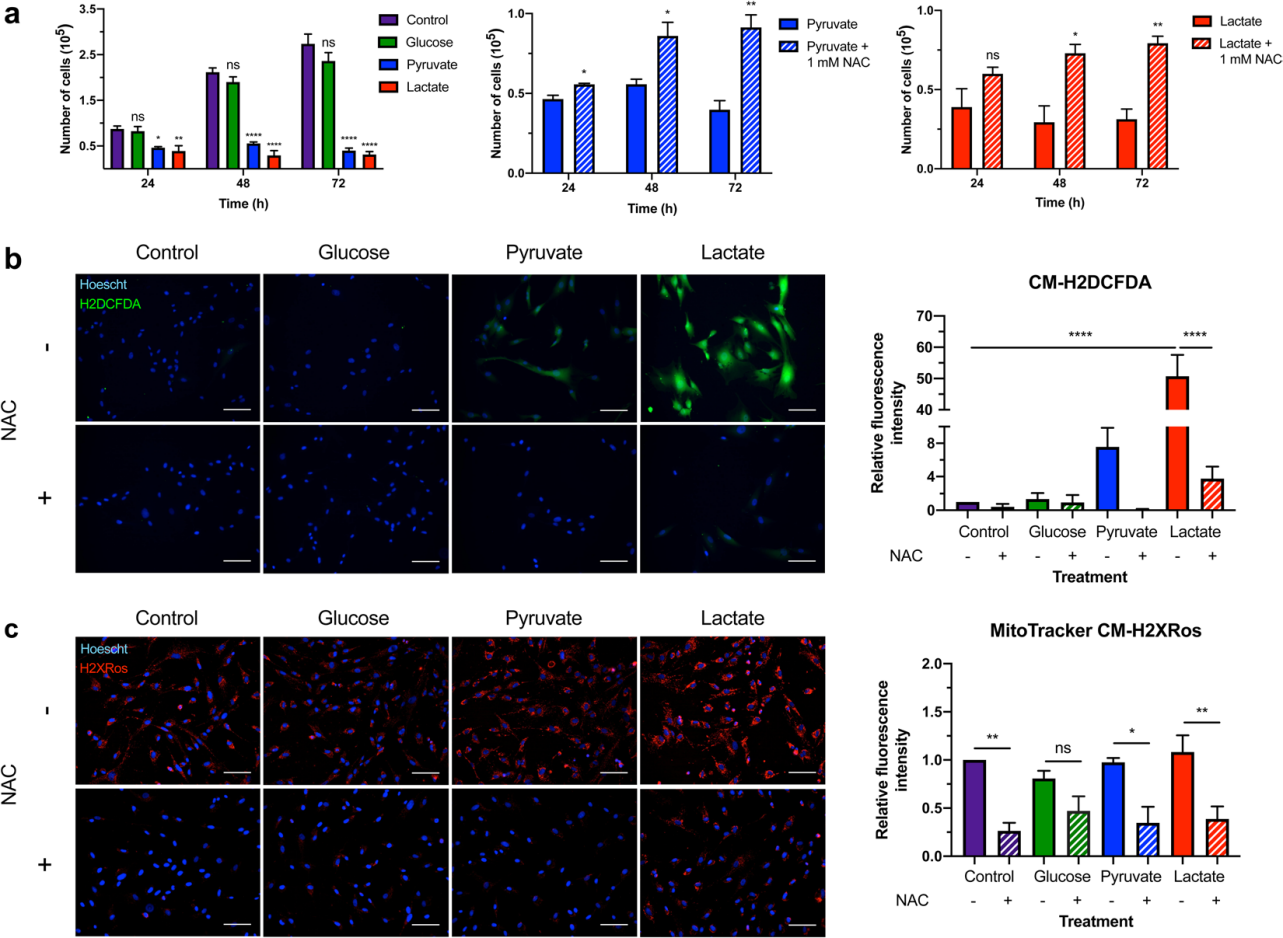
Defined metabolite treatment alters fibroblast cell growth and viability in a ROS-dependent manner. (**a**) BJ fibroblast cells were cultured in defined metabolite media for 24, 48 and 72 h. Trypan Blue exclusion was used to determine the number of live cells at each time point. After 24 h, BJ cells cultured in pyruvate or lactate medium exhibited significantly decreased cell growth, whereas glucose-treated cells exhibited no difference from control. This pattern was sustained at both 48 and 72 h *(left panel)*. Treatment with 1 mM NAC attenuated pyruvate-induced cell death at 24, 48- and 72 h post-treatment *(middle panel)* and attenuated lactate-induced cell death at 48 and 72 h post-treatment *(right panel)*. Data presented represent N = 3 ± s.e.m. with 3 technical replicates per treatment. Asterisks indicate significant difference (p < 0.05 = *, p < 0.01 = **, p < 0.0001 = ****) and ns = no difference tested by One-way ANOVA and Dunnett’s multiple comparisons test as well as an Unpaired Two-tailed student’s t-test. (**b**) BJ fibroblast cells were cultured in defined metabolite medium containing glucose, pyruvate or lactate as the sole fuel source with and without 1 mM NAC for 20 h. Live cell staining with the fluorescent cellular ROS indicator, CM-H2DCFDA (green), was performed. ImageJ analysis revealed that lactate-treated BJ cells produced significantly more ROS than all other treatments. NAC exposure significantly attenuated ROS production in lactate-treated cells. (**c**) Live cell staining with the fluorescent mitochondrial ROS indicator, MitoTracker CM-H2XRos (red), was performed. ImageJ analysis revealed that NAC significantly attenuated ROS production in control-, pyruvate-, and lactate-treated cells. A small but non-significant decrease in ROS levels was also observed in glucose-treated BJ cells supplemented with NAC. Nuclei within all cells were counterstained with Hoescht dye (blue). Scale bars = 100 μm. The fluorescence images presented are representative of at least three independent experiments. Data presented represent N = 3-4 ± s.e.m. with 3 technical replicates per treatment. Asterisks indicate significant difference (p < 0.05 = *, p < 0.01 = **, p < 0.0001 = ****) and ns = no difference tested by One-way ANOVA and Tukey’s multiple comparisons test.

In light of the observation that lactate-treated BJ fibroblasts became glycolytic upon injection with glucose and pharmacological inhibition of ATP synthase during the glycolysis stress test, we explored if this effect was sustained over a longer period. Due to the toxicity elicited by 24 h lactate treatment (Fig. 3a, *left panel)*, we set out to determine the minimum about of time BJ fibroblast cells can be cultured in lactate-only medium prior to being switched into glucose-only medium and still exhibit a metabolic shift. Fibroblasts were cultured in glucose or lactate medium for 12, 16, 20 and 24 h prior to 48 h culture in glucose medium. Immunoblot analysis of the ratio of ser^232^-PDH to total PDH was used as an indicator for glycolytic metabolism. Densitometric analysis revealed that 20 h lactate pre-treatment significantly increased the ratio of ser^232^-PDH to total PDH (p < 0.01) compared to BJ cells cultured only in glucose medium (*see* Supplementary Fig. S2).

### Defined metabolite treatment alters cell viability in a ROS-dependent manner

Glucose is the typical fuel source for most normal somatic cell types maintained *in vitro*, thus we sought to examine the impact of metabolite restriction on BJ cell growth and viability. Control- and glucose-treated cells exhibited similar growth over 72 h whereas pyruvate (p < 0.05)- and lactate-treated (p < 0.01) cells exhibited significantly decreased cell growth and elevated cell death within 24 h (Fig. 3a, *left panel*). Restricting cells to pyruvate as the sole fuel source strongly directs cellular metabolism to OXPHOS for ATP production^18^. A by-product of OXPHOS is mitochondrial ROS production^31^. While ROS are important signalling molecules, ROS build-up can cause cell death^32^. To examine if pyruvate- and lactate-induced cell death was a result of ROS build-up, the antioxidant precursor, N-acetyl-cysteine (NAC) was added to metabolite restricted media. Indeed, 24 h and 48 h of NAC exposure resulted in significantly increased viability of pyruvate- (p < 0.05) and lactate-treated (p < 0.05) fibroblast cells, respectively. (Fig. 3a*, centre and right panels*). These results suggest that pyruvate- and lactate-induced toxicity is caused, in part, by increased ROS.

To confirm that pyruvate and lactate treatment induce ROS build-up, BJ fibroblasts were cultured in defined metabolite medium for 20 h prior to live cell fluorescent quantification of ROS levels. Quantification of fluorescence intensity following staining with whole cell ROS indicator, CM-H2DCFDA, revealed that lactate-treated cells produced significantly more ROS than all other conditions (p < 0.0001) (Fig. 3b). Furthermore, NAC exposure significantly attenuated ROS production in BJ fibroblasts cultured in lactate medium (p < 0.0001) (Fig. 3b). Staining for mitochondrial ROS levels using MitoTracker CM-H2XRos revealed that mitochondrial ROS production was significantly reduced by NAC exposure in control- (p < 0.01), pyruvate- (p < 0.05) and lactate-treated (p < 0.01) BJ cells (Fig. 3c). A small but non-significant decrease in ROS levels was also observed in glucose-treated BJ cells supplemented with NAC (Fig. 3c).

### Lactate promotes increased HIF-1α protein abundance in fibroblasts in a ROS-dependent manner

**Figure 4:**
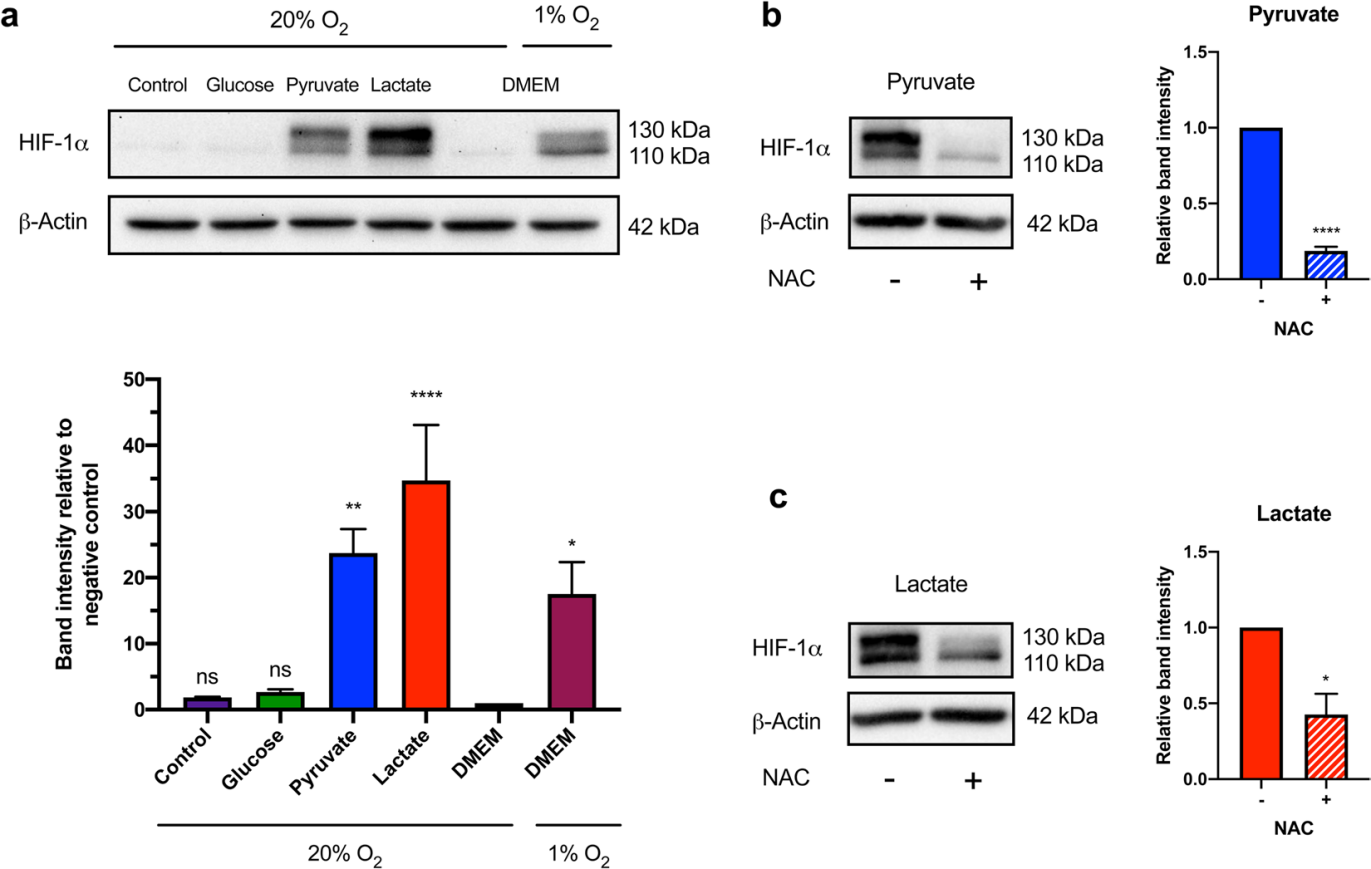
Pyruvate and lactate-treated fibroblasts exhibit increased HIF-1α stabilization under normoxic conditions, which is attenuated by NAC exposure. BJ fibroblast cells were cultured in defined metabolite medium containing glucose, pyruvate or lactate as the sole fuel source. BJ cells cultured in DMEM in normoxia (20% O_2_) or hypoxia (1% O_2_) for 20 h were used as negative and positive controls, respectively. (**a**) Immunoblot analysis revealed that HIF-1α protein levels were significantly increased in pyruvate- and lactate-treated BJ cells under normoxic conditions compared to the negative control. BJ cells were cultured in defined metabolite medium containing pyruvate or lactate as the sole fuel source with or without 1 mM NAC for 20 h in normoxic conditions. (**b**,**c**) Immunoblot analysis revealed that HIF-1α levels were lower in BJ cells treated with NAC after 20 h in both pyruvate and lactate medium. The immunoblots are representative of at least three independent experiments. Full length blots can be found in Supplementary Fig. S4. The data presented represent N = 3-5 ± s.e.m. Asterisks indicate significant difference (p < 0.05 = *, p < 0.01 = **, p < 0.0001 = ****) and ns = no significant difference tested by One-way ANOVA and Dunnett’s multiple comparison’s test and an Unpaired Two-tailed student’s t-test.

To gain mechanistic insight into the lactate-induced metabolic switch from OXPHOS to glycolysis, we examined the protein abundance of the transcription factor, HIF-1α. HIF-1α is a master regulator of glycolysis that promotes the transcription of several genes involved in glucose uptake and breakdown^38^. Under normoxic conditions, HIF-1α is translated and rapidly degraded in the cytosol^42^. Previous studies using cultured cancer cells have shown that lactate exposure results in stabilization of HIF-1α in normoxia^15^. To determine if lactate-treatment affects HIF-1α levels in non-transformed cells, fibroblasts were cultured in defined metabolite conditions for 20 h. BJ cells were cultured in DMEM under normoxic (20% O_2_) and hypoxic (1% O_2_) conditions as negative and positive controls respectively. Both pyruvate- (p < 0.01) and lactate-treated (p < 0.0001) BJ fibroblasts exhibited significantly increased HIF-1α stabilization compared to the negative control (Fig. 4a). Studies have shown that excess ROS can inhibit HIF-1α degradation under normoxic conditions^43^. To determine if lactate- or pyruvate-mediated HIF-1α stabilization was associated with ROS production, the antioxidant NAC was added to media containing only pyruvate or lactate as a fuel source. Indeed, NAC significantly attenuated HIF-1α stabilization in both pyruvate (p < 0.0001) and lactate-treated (p < 0.05) BJ cells. (Fig. 4b,c). Interestingly, NAC had a more pronounced inhibitory effect on HIF-1α stabilization in pyruvate-treated BJ cells compared to lactate-treated cells. Thus, lactate-treatment promotes stabilization of HIF-1α in BJ cells, in part, through a ROS related mechanism.

### HIF-1α accumulation primes fibroblasts to switch from OXPHOS to glycolysis by increasing the abundance of PDK1 and PKM2 proteins

**Figure 5:**
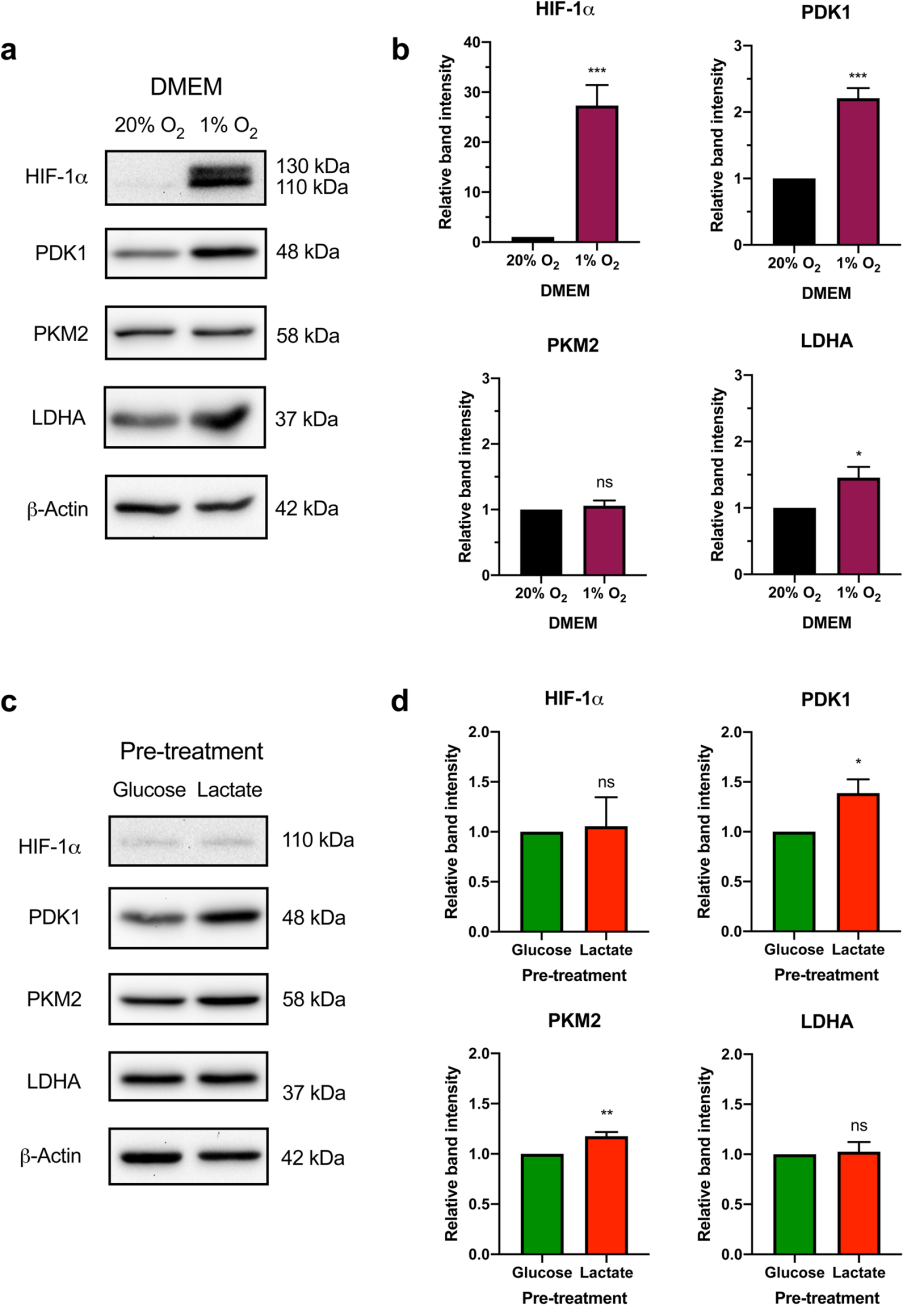
Pre-treatment of fibroblasts with lactate promotes increased protein abundance of the downstream HIF-1α targets, PDK1 and PKM2. (**a**) BJ fibroblast cells were cultured in DMEM for 20 h under either normoxic (20% O_2_) or hypoxic (1% O_2_) conditions and immunoblot analysis for the indicated downstream metabolic targets of HIF-1α was performed. (**b**) Densitometric analysis revealed that PDK1 and LDHA protein levels were significantly upregulated under hypoxic conditions, whereas PKM2 was not. (**c**) BJ cells were cultured in either glucose or lactate medium for 20 h prior to 48 h culture in glucose medium under normoxic conditions. Immunoblot analysis for the indicated downstream metabolic targets of HIF-1α was performed. (**d**) Densitometric analysis revealed that PDK1 and PKM2 protein levels were significantly upregulated in response to lactate-pre-treatment, whereas LDHA was not. The immunoblots presented are representative of four independent experiments. Full length blots can be found in Supplementary Fig. S4. The data presented represent N = 4 ± s.e.m. Asterisks indicate significant difference (p < 0.05 = *, p < 0.01 = **, p < 0.001 = ***) and ns = no difference tested by an Unpaired Two-tailed student’s t-test.

Previous studies have shown that culturing BJ cells in hypoxic conditions renders them more glycolytic^29^. To confirm the upregulation of glycolytic proteins downstream of HIF-1α, we cultured BJ fibroblasts in DMEM under normoxic (20% O_2_) and hypoxic (1% O_2_) conditions for 20 h prior to protein harvest (Fig. 5a). PDK1 (p < 0.001) and LDHA (p < 0.05) protein levels were significantly higher in BJ cells grown in hypoxia for 20 h compared to normoxic culture conditions (Fig. 5b). However, PKM2 protein levels were unaffected by hypoxia (Fig. 5b). In order to determine if the lactate-induced switch to glycolytic metabolism was sustained over longer periods, we compared the effect of pre-treating BJ cells in either glucose- or lactate-only medium for 20 h, followed by 48 h exposure to medium containing only glucose as a fuel source. We found that PDK1 (p < 0.05) and PKM2 (p < 0.01) protein levels were significantly increased in lactate pre-treated cells compared to glucose-only treated cells (Fig. 5c,d). LDHA levels did not differ between treatments (Fig. 5d).

**Figure 6:**
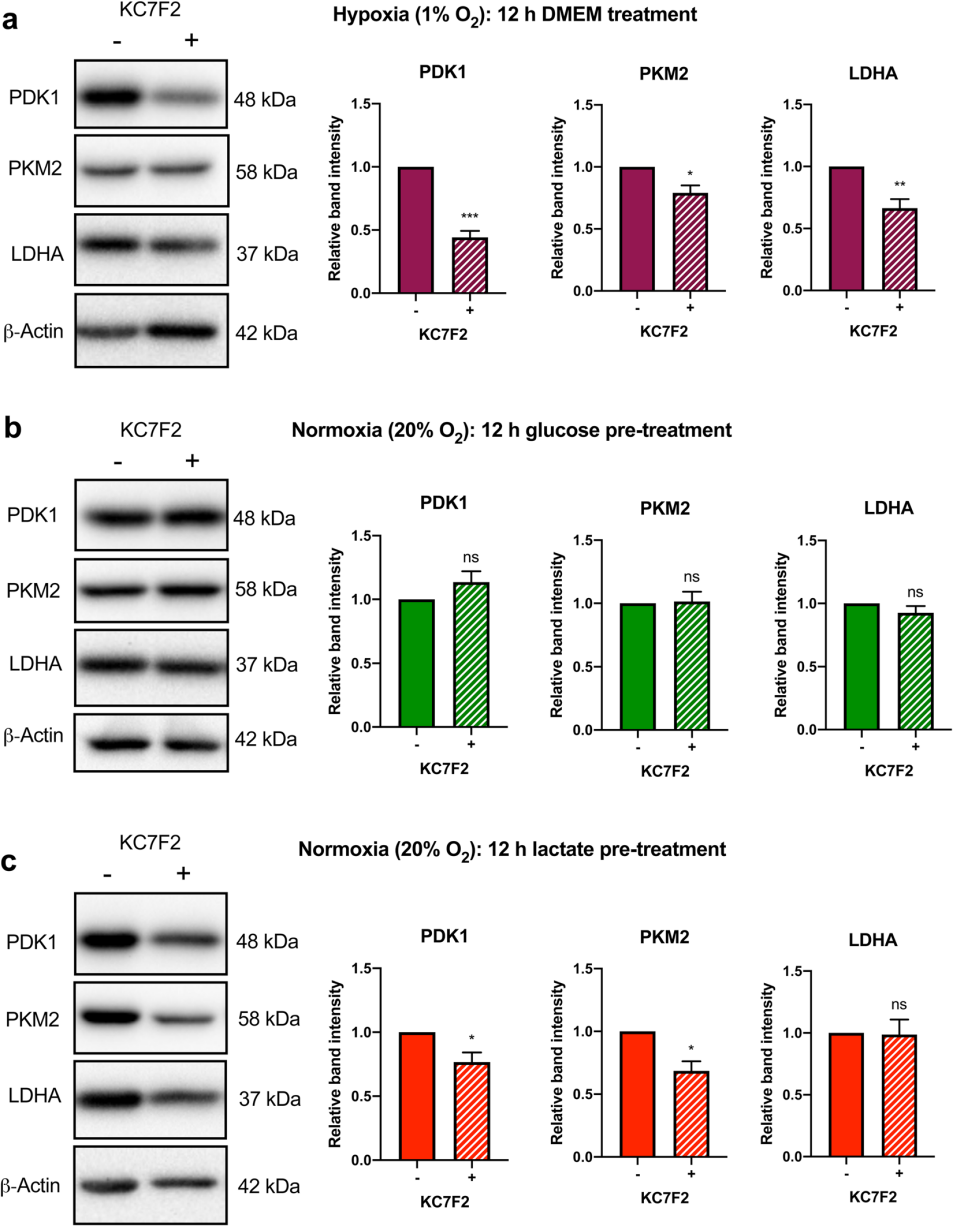
Pharmacological inhibition of HIF-1α attenuates lactate-induced upregulation of the glycolytic enzymes PDK1 and PKM2 in fibroblasts. To confirm that HIF-1α regulates PDK1, PKM2 and LDHA, BJ fibroblast cells were cultured in DMEM for 12 h under hypoxic (1% O_2_) conditions with and without the HIF-1α inhibitor, KC7F2. (**a**) Immunoblot analysis of the indicated downstream metabolic targets of HIF-1α was performed. Densitometric analysis revealed that PDK1, PKM2 and LDHA protein levels were significantly downregulated in the presence of KC7F2 under hypoxic conditions. To determine if lactate pre-treatment upregulates PDK1 and PKM2 in a HIF-1α dependent manner, BJ cells were cultured in either glucose or lactate medium for 12 h with and without the HIF-1α inhibitor, KC7F2, prior to 48 h culture in glucose medium under normoxic (20% O_2_) conditions. Immunoblot analysis of downstream metabolic target of HIF-1α was performed. (**b**) Densitometric analysis revealed that PDK1, PKM2 and LDHA protein levels were unaffected by KC7F2 in BJ cells pre-treated with glucose. (**c**) However, BJ cells pre-treated with lactate exhibited significantly lower protein levels of PDK1 and PKM2 in the presence of KC7F2 compared to lactate pre-treatment alone. The immunoblots presented are representative images of three independent experiments. Full length blots can be found in Supplementary Fig. S4. The data presented represent N = 3 ± s.e.m. Asterisks indicate significant difference (p < 0.05 = *, p < 0.01 = **, p < 0.001 = ***) and ns = no significant difference tested by and Unpaired Two-tailed student’s t-test.

To confirm that elevated PDK1 and PKM2 protein levels observed in the lactate pre-treated cells were related to HIF-1α stabilization, BJ cells were cultured in glucose or lactate medium with and without KC7F2, a pharmacological inhibitor of HIF-1α^44^. Due to the combined toxicity of KC7F2 and lactate, BJ fibroblasts were only cultured in lactate medium supplemented with 20 μM KC7F2 for 12 h instead of 20 h. We confirmed that 12 h treatment with 20 μM KC7F2 was sufficient to significantly reduce HIF-1α protein levels in BJ cells (p < 0.01) (*see* Supplementary Fig. S3). Upregulation of PDK1 (p < 0.001), PKM2 (p < 0.05) and LDHA (p < 0.01) induced by 12 h culture in hypoxic conditions was significantly attenuated by KC7F2 treatment (Fig. 6a). In contrast, PDK1, PKM2 and LDHA protein levels were unchanged in fibroblasts pre-treated with glucose medium in the presence of KC7F2 for 12 h (Fig. 6b). However, PDK1 and PKM2 levels were significantly lower (p < 0.05) in fibroblasts pre-treated with lactate in the presence of KC7F2 for 12 h (Fig. 6c). These findings support our claim that lactate pre-treatment primes BJ fibroblast cells to upregulate glycolytic enzymes in a HIF-1α-dependent manner.

### Lactate preconditioning upregulates the transcript abundance of *MYC* and *SNAI1*

**Figure 7:**
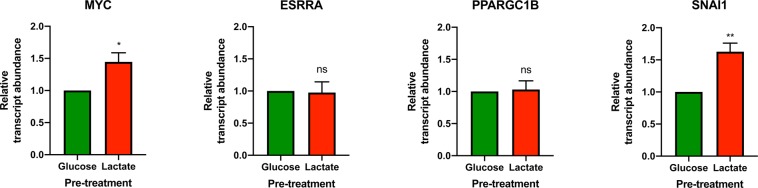
Pre-treatment of fibroblasts with lactate promotes increased transcript abundance of *MYC* and *SNAI1*. BJ fibroblast cells were cultured in either glucose or lactate medium for 20 h followed by 48 h culture in glucose medium under normoxic conditions prior to RNA extraction. qRT-PCR analysis using *HPRT1* and *RPL37A* as housekeeping genes revealed that *MYC* and *SNAI1* transcript abundance was significantly increased in response to lactate pre-treatment. In contrast, *ESRRA* and *PPARGC1B* transcript abundance were no different between treatments. The data presented represent N = 3 ± s.e.m. with 3 technical replicates per treatment. Asterisks indicate significant difference (p < 0.05 = *, p < 0.01 = **) and ns = no significant difference tested by an Unpaired Two-tailed student’s t-test.

Recent studies have demonstrated that c-MYC promotes a hyperenergetic state during early reprogramming to facilitate optimal iPSC generation^27^. Estrogen related receptor alpha (ERRα) and it’s cofactor, peroxisome proliferator-activator receptor gamma coactivator 1-beta (PGC1-β), are also implicated in the acquisition of this hyperenergetic state^36^. In addition to a metabolic switch, somatic cells must undergo a mesenchymal-to-epithelial transition during reprogramming^45,46^. Although snail family transcriptional repressor 1 (SNAIL) is a mediator of epithelial-to-mesenchymal transition (EMT), it is paradoxically essential to early somatic cell reprogramming^45,46^. To gauge the impact of lactate pre-treatment on these markers of early reprogramming, BJ cells were cultured in glucose or lactate medium for 20 h prior to 48 h cultivation in glucose-only medium. Significantly increased transcript abundance of both *MYC* (p < 0.05) and *SNAI1* (p < 0.01), but not *ESRRA* or *PPARGC1B*, was observed in lactate pre-treated fibroblast cells compared to cells cultured only in glucose medium (Fig. 7). These findings suggest that lactate production may regulate expression of specific genes involved in early somatic cell reprogramming.

## Discussion

In this study, we demonstrated that pre-treating human fibroblast cells with culture medium containing lactate as the sole fuel source, facilitates a metabolic switch from OXPHOS to glycolysis, in part, through ROS-mediated stabilization of HIF-1α. Specifically, we observed that BJ fibroblasts cultured in medium containing lactate or pyruvate as a fuel source for 24 h exhibited significantly reduced phosphorylation of PDH. Conversely, fibroblasts cultured in glucose-containing medium for 24 h displayed elevated PDH phosphorylation, a marker of glycolytic metabolism^38,47^. These findings are consistent with a previous study in which HeLa cells grown under glucose-only culture conditions exhibited reliance on aerobic glycolysis^18^. However, when cultured in pyruvate-only medium, HeLa cells switched to OXPHOS to facilitate ATP production^18^. Interestingly, despite glucose deprivation, lactate-treated BJ cells unexpectedly exhibited increased transcript abundance of several genes encoding proteins responsible for catalysing steps of the glycolytic pathway. A recent study by Zhang *et al*. discovered that lactate acts as an epigenetic regulator by inducing histone lactylation in a dose-dependent manner^48^. Indeed, the onset of aerobic glycolysis or hypoxia-induced glycolysis directly correlated with both increased lactate production and histone lactylation, and direct induction of glycolytic gene expression^48^. Therefore, it is possible that in our model, lactate medium transcriptionally primes human fibroblasts for glycolytic metabolism pending substrate availability. Earlier studies have demonstrated that cancer cells are capable of using lactate as their preferred fuel source^14,17^. Furthermore, a study by Hui *et al*. revealed that lactate is the primary fuel source for the TCA cycle in most tissues and tumours^13^.

Metabolic flux, as assessed by Seahorse analysis, further supported our theory that lactate treatment primes BJ cells for glycolytic metabolism. BJ fibroblasts pre-treated with lactate medium exhibited a significantly greater glycolytic reserve and significantly lower spare respiratory capacity compared to fibroblasts pre-treated with pyruvate medium. Recent studies using primary human dermal fibroblasts showed that mitochondrial spare respiratory capacity negatively correlates with somatic cell reprogramming efficiency as well as pluripotency^37,49^. Interestingly, we found that lactate pre-treatment resulted in greater BJ cell basal respiration compared to pyruvate pre-treatment. Elevated basal respiration and reduced spare respiratory capacity in lactate pre-treated fibroblasts implies that these cells were respiring at their maximum capacity even prior to induced ETC uncoupling. c-MYC induces a hyperenergetic metabolic state during reprogramming that is necessary for the transition to pluripotency^27^. Our findings demonstrate that while both pyruvate and lactate treatment result in reduced PDH phosphorylation, only lactate pre-treatment promotes a hyperenergetic bivalent metabolic state.

Although BJ fibroblasts were capable of using lactate or pyruvate as fuel sources, both metabolites promoted an inhibition of cell growth and elevated cell death. Cell growth is maintained by the production of anabolic precursors such as ribose-5-phosphate (R5P), in a manner largely dependent on the pentose phosphate pathway (PPP)^50,51^. In addition to providing R5P, the PPP also produces nicotinamide adenine dinucleotide phosphate (NADPH), a key metabolic product that provides the reducing power to fuel protein-based antioxidant systems and recycle oxidized glutathione^51^. Since PPP activity relies on glycolytic flux, it is possible that restricting fibroblasts to lactate or pyruvate as a fuel source results in a deprivation of vital upstream glycolytic intermediates within the PPP that would otherwise support proliferation and antioxidant systems. Supplementation with NAC, a precursor to the antioxidant, glutathione, improved the viability of pyruvate- and lactate-treated BJ fibroblast cells. These findings suggest that pyruvate and lactate treatments elicit cytotoxicity via oxidative stress caused by excess ROS production and/or insufficient antioxidant synthesis^52^.

While peroxisomes and the endoplasmic reticulum are organelles capable of generating cellular ROS, mitochondria are the major site of ROS production in mammalian cells^53^. ROS exit the mitochondria and enter the cytosol by diffusion or passage through voltage dependent anion channels (VDAC)^54^. Under oxidative stress conditions, a growing body of work has revealed that mitochondrial ROS activates transient openings of voltage-gated mitochondrial permeability transition pore (mPTP) channels^55,56^. When open, matrix metabolites, such as ROS, exit the mitochondria through mPTPs and enter the cytosol^54–56^. This effect has been coined ROS-induced ROS release (RIRR)^55,57^. Cytosolic ROS has the potential to react with redox-sensitive molecules, activate redox-sensitive signalling pathways and induce RIRR in proximal mitochondira^55^. For example, during the Fenton reaction, hydrogen peroxide (H_2_O_2_) reacts with ferrous iron (Fe^2+^) to generate ferric iron (Fe^3+^), a hydroxyl radical (•OH), and a hydroxyl ion (OH^−^)^58^. Transient ROS-induced mPTP openings are associated with early phase somatic cell reprogramming and their metabolic switch^35,59^. Studies in human and mouse fibroblast cells have shown that mPTP openings promote demethylation of pluripotency promoters, an integral event in the acquisition of pluripotency during the later stages of reprogramming^35,59^. Indeed, increasing mPTP opening frequency with ROS-inducing agents prior to the metabolic switch was shown to increase reprogramming efficiency^35^. Our findings demonstrate that while pyruvate- and lactate-treated fibroblasts do not differ in their production of mitochondrial ROS after 20 h, lactate-treated cells exhibit significantly greater total cellular ROS levels. It is possible that lactate promotes mPTP openings, accelerating the release of ROS into the cytosol. Furthermore, lactate has been shown to propagate ROS production *via* the Fenton reaction by forming a complex with Fe^3+^ that reacts with H_2_O_2_ to produce additional •OH^60^. As such, we are currently investigating the impact of lactate medium on ROS-induced mPTP openings.

During early somatic cell reprogramming, elevated OXPHOS promotes a ROS burst which subsequently activates the transcription factor Nuclear factor erythroid 2-related factor 2 (NRF2) which, in turn, promotes increased transcription of *HIF-1α*^34^. HIF-1α facilitates increased glycolytic metabolism by upregulating transcription of genes encoding glycolytic enzymes such as PDK1, PKM2 and LDHA^37^. Under normoxic conditions, HIF-1α is rapidly synthesized and degraded in the cytosol^42^. HIF-1α degradation is mediated by prolyl hydroxylase domain protein (PHD), which hydroxylates HIF-1α, recruiting the von Hippel Lindau (VHL) complex^15^. Once bound, VHL poly-ubiquitinates HIF-1α, tagging it for proteasomal degradation^15^. PHD requires the cofactors O_2_, Fe^2+^, and α-ketoglutarate (α-KG)^61^. Under hypoxic conditions, insufficient O_2_ renders PHD inactive, permitting HIF-1α to escape degradation, accumulate in the cytosol, and translocate to the nucleus where it facilitates transcription^15^.

As many tumours exist in hypoxic environments, it is not surprising that they exhibit high levels of HIF-1α^62^. However, various cellular conditions exist which allow for HIF-1α accumulation in normoxia^15,43,63^. For example, ROS stabilizes HIF-1α under normoxic conditions by oxidizing Fe^2+^ to Fe^3+^, thereby rendering PHD inactive^64,65^. Further evidence has emerged that lactate can inhibit PHD activity through its conversion to pyruvate which competitively inhibits α-KG from associating with PHD^15^. In this study we showed that both pyruvate and lactate are capable of stabilizing HIF-1α protein levels under atmospheric oxygen. However, NAC treatment only partially attenuated lactate-induced HIF-1α stabilization whereas pyruvate-induced HIF1α stabilization was almost entirely ablated by NAC exposure. These findings suggest that while both pyruvate and lactate can facilitate HIF-1α stabilization through ROS production, lactate may further directly stabilize HIF-1α in a ROS-independent manner. It is also possible that lactate-induced histone lactylation indirectly contributes to lactate-mediated HIF-1α stabilization in BJ fibroblast cells. Although these are plausible mechanisms to explain our finding that exogenous lactate increases the transcript abundance of genes encoding several glycolytic enzymes, further studies are warranted to explore this relationship.

**Figure 8:**
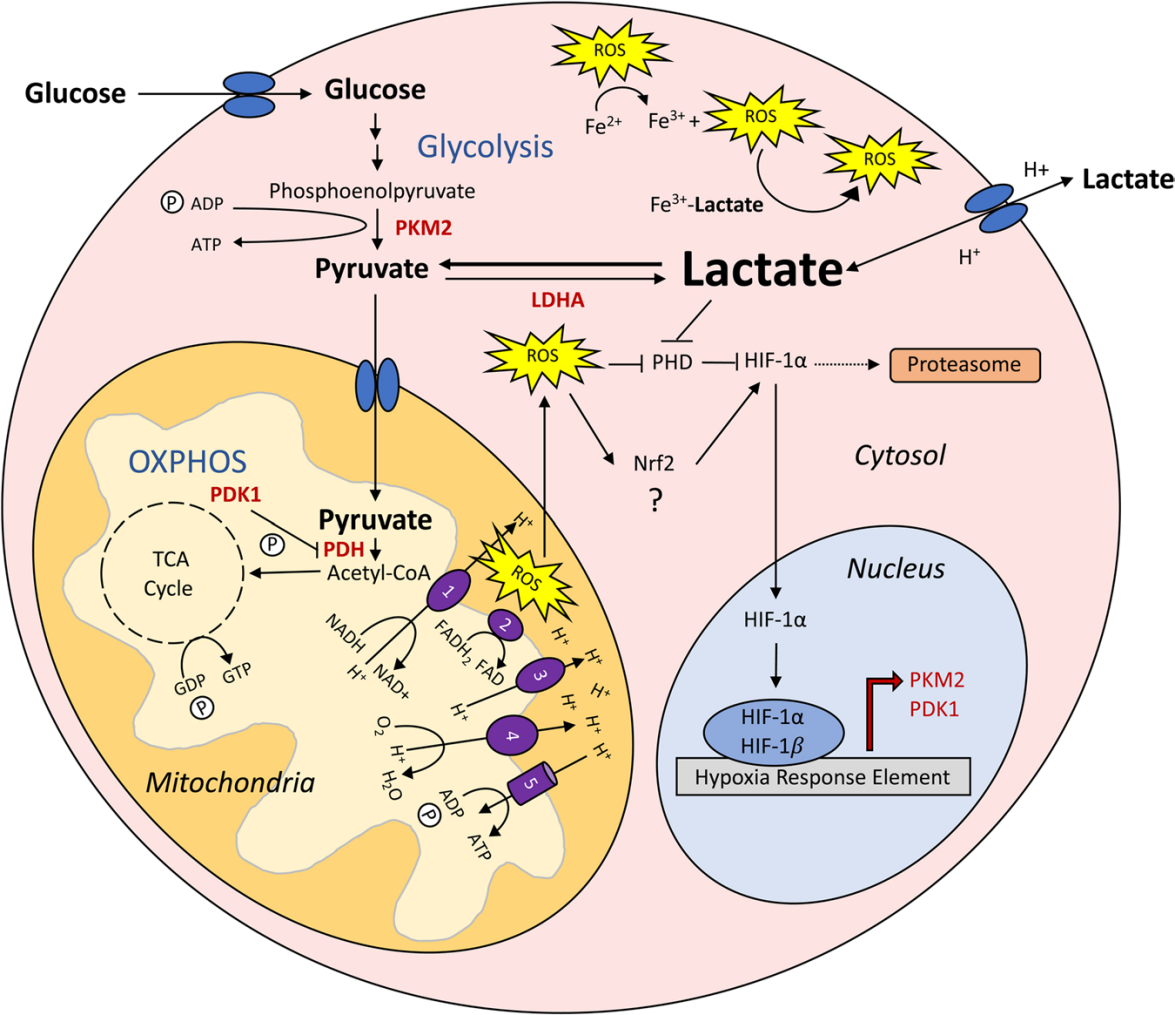
Proposed mechanism of action for lactate-induced upregulation of glycolytic metabolism in normal human diploid fibroblasts. Extracellular lactate is taken up by monocarboxylic acid transporters and can then be converted to pyruvate to fuel the tricarboxylic acid (TCA) cycle, which produces reducing agents that support electron transport chain (ETC)-mediated adenosine triphosphate (ATP) production by oxidative phosphorylation (OXPHOS). The mitochondrial reactive oxygen species (ROS) produced by ETC activity translocate to the cytosol freely, through voltage dependent anion channels (VDAC), or through open mitochondrial permeability transition pores. In the cytosol ROS inhibit prolyl hydroxylase (PHD) activity through the Fenton reaction which oxidizes ferrous iron (Fe^2+^), a critical PHD cofactor, to ferric iron (Fe^3+^). Following the inactivation of PHD, hypoxia inducible factor 1 alpha (HIF-1α) is no longer tagged for proteasomal degradation. Instead, HIF-1α translocates to the nucleus where it dimerizes with HIF-1β and binds to the hypoxia response element (HRE) initiating the transcription of glycolytic enzymes such as pyruvate dehydrogenase kinase 1 (PDK1) and pyruvate kinase muscle isozyme 2 (PKM2). Lactate is also capable of inhibiting PHD activity through its conversion to pyruvate which competitively inhibits PHD co-factor, α-ketoglutarate (α-KG), from associating with PHD. Lactate may also enhance ROS-mediated inhibition of PHD by the ROS induced ROS release (RIRR) effect. Lactate can form a complex with Fe^3+^ which then reacts with the ROS generated from the Fenton reaction to propagate the production of more ROS. It is also possible that lactate-mediated ROS production promotes increased HIF-1α levels by activating nuclear factor erythroid 2-related factor 2 (NRF2). However, future studies are required to elucidate the role of NRF2 in lactate-mediated induction of glycolytic metabolism.

In this study we showed that lactate pre-treatment significantly increased PDK1 and PKM2 protein levels in BJ cells through a HIF-1α-dependent mechanism. Small molecule activation of HIF-1α during somatic cell reprogramming has been shown to dramatically increase fibroblast reprogramming efficiency by upregulating PDK1 and PKM2^38^. It is possible that lactate promotes HIF-1α stabilization by inhibiting PHD activity through competitive inhibition of α-KG and by propagating the Fenton reaction (Fig. 8). Furthermore, as NRF2 has been shown to act upstream of HIF-1α, it is possible that lactate-mediated ROS production initiates the NRF2 cell stress response pathway to further upregulate HIF-1α^34^ (Fig. 8). However, further studies are warranted to discern the impact of lactate treatment on NRF2 activity in BJ fibroblasts.

In addition to lactate pre-treatment promoting increased PKM2 and PDK1 protein abundance, we demonstrated that this pre-treatment strategy results in increased transcript abundance of *SNAI1* and *MYC*. Elevated *SNAI1* transcript abundance following lactate exposure is in line with a study conducted in lung cancer cells which demonstrated that that lactate promotes *SNAI1* expression in a dose-dependent fashion^66^. With respect to *MYC*, recent focus has shifted towards its endogenous role. Exogenous expression of *MYC* is considered a dispensable Yamamaka factor that can be replaced by overexpression of *PDK1* and *PKM2*, various chemicals, or other enhancer factors such as *NANOG* and *LIN28*^25,26,30,38^. Prieto *et al*. demonstrated that endogenous c-MYC is fundamental to early stage reprogramming events such as mitochondrial remodelling and activation of glycolysis^27^. It is therefore possible that the lactate-induced metabolic shift from OXPHOS to glycolysis observed in this study is in part mediated by endogenous c-MYC. Interestingly, other markers for early stage reprogramming, including *ESRRA* and *PPARGC1B*, where not affected by lactate exposure. It is possible that lactate exposure promotes lactylation of specific histone lysine residues and selectively induces expression of certain pluripotency genes. Future studies using chromatin immunoprecipitation with anti-lactyllysine antibodies^48^ will help identify genes regulated by histone lactylation following exposure of human fibroblasts to exogenous lactate.

Mesenchymal to epithelial transition (MET) is another hallmark of reprogramming fibroblasts to iPSCs^45,46^. Counterintuitively, Unternaehrer *et al*. demonstrated that human and mouse fibroblasts cells expressing higher levels of endogenous *SNAI1*, an EMT regulator, actually exhibited more efficient reprogramming^45^. These researchers further postulated that *SNAI1* expression increases reprogramming efficiency by inhibiting let-7 family members^45^. Let-7 is a family of tumour suppressors whose inhibition has been shown to promote reprogramming efficiency^67^. LIN28, a regulator of stem cell metabolism, is a known repressor of let-7 miRNA processing^68,69^. It is therefore possible that lactate pre-treatment has the potential to increase reprogramming efficiency not only by promoting the metabolic switch event, but also by mirroring LIN28-mediated repression of let-7 miRNA processing through increased *SNAI1* expression.

In conclusion, our work demonstrates that short-term culturing of normal human dermal fibroblast cells in medium containing lactate as the sole metabolite fuel source primes BJ fibroblast cells to transition from OXPHOS to glycolysis metabolism. Indeed, fibroblasts cultured in lactate medium exhibit increased ROS production which, in part, contributes to the stabilization of HIF-1α and subsequent upregulation of glycolytic enzymes, PDK1 and PKM2. By promoting the transition from OXPHOS to glycolysis, lactate pre-treatment could serve as a novel approach to amplify the metabolic switch during the generation of iPSCs. Furthermore, by triggering HIF-1α-mediated upregulation of PDK1 and PKM2 as well as increased transcript abundance of *MYC* and *SNAI1*, lactate treatment may be able to eliminate the need for exogenous c-MYC during somatic cell reprogramming. We are currently in the process of exploring these hypotheses. Ultimately, the findings from this study may lead to the development of a safer and more efficient method of creating human iPSCs that can be utilized for pluripotent stem cell-based therapies.

## Materials and Methods

### Cell culture

The normal, diploid (46, XY) BJ fibroblast cell line (ATCC CRL-2522) was purchased from the American Type Culture Collection. This cell line was maintained in Dulbecco’s Modified Eagle’s Medium (DMEM) (#319-005-CL; Wisent) supplemented with 10% fetal bovine serum (FBS) (#c17-513F; Gibco) and 1% penicillin and streptomycin (#15140122; Gibco) at 5% CO_2_, 37 °C. To generate a positive control for HIF-1α experiments, fibroblasts were maintained in the above culture medium and incubated in a HypoxyLab (Oxford Optronix) chamber at 5% CO_2_, 37 °C, 1% O_2_.

Defined metabolite media were prepared as follows. Base medium was prepared by dissolving DMEM powder lacking glucose, L-glutamine, sodium pyruvate and sodium bicarbonate (#5030; Sigma-Aldrich) in 1 L deionized/double distilled water supplemented with 3.7 g/L sodium bicarbonate (#SX0320-1; EMD Millipore) and sterilized through a 0.1 μm filter. Immediately prior to experimentation, base media was supplemented with 4 mM L-glutamine (#17605-E; Lonza) and 10% FBS. FBS was dialyzed using regenerated cellulose dialysis tubing with a 3,500 Dalton cut-off (#21-152-9; Fisher Scientific) for 2 d in buffered base medium with one media change and sterilized through a 0.2 μm filter. Control, glucose, pyruvate and lactate defined metabolite media were prepared by adding 20 mM D-(+)-glucose (#G7021; Sigma-Aldrich) and 1 mM sodium pyruvate (#P2256; Sigma-Aldrich), 20 mM glucose, 20 mM sodium pyruvate and 20 mM sodium L-lactate (#71718; Sigma-Aldrich), respectively, to the supplemented base medium. A metabolite concentration of 20 mM was selected based on reports that the concentration of lactate in tumour microenvironments most commonly lies within the range of 10–30 mM^10,70^. Prior to treatment with defined metabolite medium, fibroblasts were washed twice with phosphate buffered saline (PBS) (#17-513F; Lonza) to remove traces of DMEM.

To determine if BJ fibroblasts were producing ROS as a result of their treatment, the antioxidant, N-acetyl-L-cysteine (NAC) (#A7250; Sigma-Aldrich), was added at a concentration of 1 mM to cultures. NAC was prepared as a 0.5 M stock solution in buffered base medium and stored at 4 °C.

### Immunoblot analysis

BJ fibroblasts were washed twice in PBS and lysed in ice-cold RIPA buffer (10 mM Tris-HCl pH 8.0, 1% Triton-X-100, 0.1% Sodium deoxycholate, 0.5 mM EGTA, 0.1% SDS, 140 mM NaCl) containing 0.5X Halt Protease Inhibitor Cocktail (100×) (#1862209; Thermo Scientific), 0.5X EDTA (100×) (#1861274; Thermo Scientific), 1X phenolmethanesulfonyl fluoride (PMSF) (#P7626; Sigma-Aldrich) and 1X sodium orthovanadate (NaOV) (#S6508; Sigma-Aldrich) for 15 min on ice prior to the removal of cellular debris by centrifugation at 14,800 rpm for 10 min at 4 °C. Following determination of protein concentration using a DC protein assay (Bio-Rad), protein extracts (15–20 µg) were resolved by 10% SDS-PAGE, then electroblotted onto polyvinylidene fluoride (PVFD) membranes (Bio-Rad) and blocked in TBS-T containing 3% bovine serum albumin (#0331; VWR) and 1% non-fat dry milk (#9999 S; Cell Signalling). The following primary antibodies were used: pSER^232^-PDH (#AP1063; EMD Millipore); PDH (#ab110334; ABCAM); PDK1 (#ADI-KAP-PK112-f; Enzo Life Science); PKM2 (#3198; Cell Signalling); LDHA (#2012; Cell Signalling); HIF-1α (#ab179483; ABCAM) and β-Actin (#sc-47778; Santa Cruz). Following overnight incubation with primary antibodies, PVDF membranes were incubated with HRP-conjugated secondary mouse (sc-2006; Santa Cruz) and rabbit (#sc-2006; Santa Cruz) antibodies at a 1:10,000 dilution. Bands were detected using SuperSignal West Pico chemiluminescence substrate (#34080; Thermo Scientific), Luminata Classico chemiluminescence substrate (#WBLUC0500; EMD Millipore), or Luminata Forte chemiluminescence substrate (#WBLUF0500; EMD Millipore). Immunoblots were imaged using a Chemidoc XRS System (Bio-Rad). Band density quantification was performed using Image Lab software version 5.2 (Bio-Rad).

The following modifications were made for immunoblot analysis of HIF-1α. BJ fibroblasts were washed once in ice-cold PBS and lysed in ice-cold Triton-X-100 buffer (20 mM Tris-HCl pH 8.0, 150 mM NaCl, 10% glycerol, 1% Triton-X-100, 6 mM MgCl_2_) containing 0.5X Halt Protease Inhibitor Cocktail (100×), 0.5X EDTA (100×), 1X PMSF, and 1X NaOV for 15 min on ice followed by centrifugation to remove cellular debris at 14,800 rpm at 4 °C for 10 min. Sample preparation steps did not differ from above protocol. For the HIF-1α positive control, all steps up to the 15 min lysis in Triton-X-100 buffer were performed in the HypoxyLab (Oxford Optronix). Samples of 20-30 μg protein were resolved by 10% SDS-PAGE, electroblotted onto PVDF membranes and processed as described above.

### RNA extraction and real time PCR analysis

RNA was isolated from BJ fibroblasts using Trizol and chloroform extraction. RNA concentration was quantified using a Nanodrop 2000 (Thermo Scientific) and all samples underwent DNase treatment using a DNase Treatment Kit (#AMP D1; Sigma) prior to reverse transcription using Moloney’s-Murine Leukemia Virus Reverse Transcriptase (M-MLV) Kit (#28025-021; Life Technologies) with random primers (#C1181; Promega) and dNTP’s (#R0181; Life Technologies). TaqMan Gene Expression Assays (Life Technologies) and TaqMan Fast Advanced Master Mix (#4444557; Life Technologies) were used to facilitate amplification as per the manufacturer’s directions. TaqMan Gene Expression Assay details can be found in Supplementary Table S1. The threshold cycle (Ct) value was determined using a CFX96 Touch Real-Time PCR Detection System (Bio-Rad). Relative transcript abundance (ΔΔCq) was calculated using CFX Maestro 1.1 software version 4.1.2433.1219 (Bio-Rad). Transcript abundance was normalized against the geometric mean of two of the following housekeeping genes, *ACTB, HPRT1* and *RPL37A*. Housekeeping genes were determined using Human Endogenous Control Panels (#4426696; Applied Biosystems).

### Seahorse XF^e^24 flux analysis

BJ fibroblasts were seeded on XF^e^24 microplates (Agilent) coated with 50 μg/mL poly-D-lysine/well in control, glucose, pyruvate or lactate defined metabolite medium at the following densities: 45,000 cells/well (control), 50,000 cells/well (glucose), 68,000 cells/well (pyruvate), 53,000 cells/well (lactate).

Prior to the glycolysis stress test, fibroblasts were washed twice in sodium bicarbonate-free XF Base Medium (#102353-100; Agilent) supplemented with 4 mM L-glutamine at a pH of 7.35+/−0.05 and incubated in the assay medium for 1 h at 37 °C, 0% CO_2_. Basal extracellular acidification rate (ECAR) was determined following injection of 10 mM glucose. 1 μM Oligomycin and 50 mM 2-Deoxy-D-glucose (2-DG) were sequentially injected to determine glycolytic capacity and glycolytic reserve. All injections were provided in a Seahorse XF Glycolysis Stress Test Kit (#103020-100; Agilent).

Prior to the Mitochondrial Stress Tests, fibroblasts were washed twice in sodium bicarbonate-free XF Base Medium pH 7.35+/−0.05, supplemented with 4 mM L-glutamine, 10 mM glucose, and 1 mM sodium pyruvate and incubated in assay medium for 1 h at 37 °C, 0% CO_2_. Oxygen consumption rate (OCR) was first measured at baseline. Following sequential injection of 1 μM Oligomycin, 1 μM FCCP and 0.5 μM antimycin A/rotenone, maximal respiration and spare respiratory capacity were determined. All injections were provided in a Seahorse XF Cell Mito Stress Test Kit (#103015-100; Agilent). ECAR and OCR were normalized to total protein by lysing cells using RIPA buffer and quantifying protein content using a DC protein assay (Bio-Rad). Wave Desktop software version 2.4.1, Mitochondrial Stress Test Report Generator version 3.0.5 and Glycolysis Stress Test Report Generator version 3.0.6 were used to process data.

### Cell viability assay

BJ fibroblasts were seeded at 53,000 cells/well in 12-well dishes in DMEM and allowed to adhere overnight. Cells were then washed twice with PBS and subjected to either control, glucose, pyruvate or lactate treatment +/−1 mM NAC. The number of live cells/well was counted using Trypan blue (#17-942E; Lonza) dye exclusion every 24, 48, and 72 h after exposure to defined metabolite media. Cells were counted using a haemocytometer viewed under a Leica phase contrast microscope using a 10X objective.

### Fluorescence microscopy

BJ fibroblasts were cultured in defined metabolite media for 20 h +/−1 mM NAC prior to live cell fluorescence imaging. To detect whole cell ROS, cells were washed twice with PBS and stained with 2.5 μM CM-H2DCFDA (#C6827; ThermoFisher) in serum-free defined metabolite media for 15 min at 5% CO_2_, 37 °C. To detect mitochondrial ROS, Cells were washed twice with PBS and stained with 200 nM MitoTracker CM-H2XRos (#M7513; ThermoFisher) in serum-free defined metabolite media for 20 min at 5% CO_2_, 37 °C. Following incubation, BJ cells were washed with PBS and stained with 10 μg/mL Hoechst (#H3570; Life Technologies) for 1 min at room temperature. Fibroblasts were washed once more in PBS and imaged in phenol red-free defined metabolite media at room temperature using a Zeiss Axio Observer A1 microscope. Digital images were acquired using a Q-Imaging Retiga camera and processed using ImageJ software version 2.0.0-rc-43/1,50e.

### Pharmacological agents

The HIF-1α inhibitor, KC7F2 (#4324; TOCRIS) was dissolved in DMSO (#D8418; Sigma-Aldrich) to obtain a stock concentration of 50 mM. BJ fibroblasts were cultured in defined metabolite media or DMEM containing 20 μM KC7F2 under either hypoxic (1% O_2_) or normoxic (20% O_2_) conditions for 12 h to induce inhibitory effects.

### Statistical analysis

All data presented represent the mean ± s.e.m. of at least three biological replicates. Difference between three or more means was assessed using One-way ANOVA and Dunnett’s multiple comparisons or Tukey’s multiple comparisons test. Difference between two means was assessed by an Unpaired Two-tailed student’s t-test in GraphPad Prism version 8.1.2. The difference between means was deemed significant at p < 0.05. The ROUT method was used to identify outliers among biological replicates (with Q set to 1%).

## Supplementary information

Supplementary information.

## Data Availability

All data generated or analysed during this study are included in this published article.

## References

[1] Warburg O, Wind F, Negelein E (1927). The metabolism of tumors in the body. J. Gen. Physiol..

[2] Cori CF, Cori GT (1925). The carbohydrate metabolism of tumors. J. Biol. Chem..

[3] Polet F, Feron O (2013). Endothelial cell metabolism and tumour angiogenesis: glucose and glutamine as essential fuels and lactate as the driving force. J. Intern. Med..

[4] Fu Y (2017). The reverse Warburg effect is likely to be an Achilles’ heel of cancer that can be exploited for cancer therapy. Oncotarget.

[5] Lee M, Yoon J (2015). Metabolic interplay between glycolysis and mitochondrial oxidation: The reverse Warburg effect and its therapeutic implication. World J. Biol. Chem..

[6] Migneco G (2010). Glycolytic cancer associated fibroblasts promote breast cancer tumor growth, without a measurable increase in angiogenesis: Evidence for stromal-epithelial metabolic coupling. Cell Cycle.

[7] Mikkilinei L (2017). Hodgkin lymphoma: a complex metabolic ecosystem with glycolytic reprogramming of the tumor microenvironment. Semin. Oncol..

[8] Pértega-Gomes N (2014). A lactate shuttle system between tumour and stromal cells is associated with poor prognosis in prostate cancer. BMC Cancer.

[9] Arcucci A, Ruocco MR, Granato G, Sacco AM, Montagnani S (2016). Cancer: An oxidative crosstalk between solid tumor cells and cancer associated fibroblasts. Biomed Res. Int..

[10] Pérez-Escuredo J (2016). Lactate promotes glutamine uptake and metabolism in oxidative cancer cells. Cell Cycle.

[11] Panisova E (2017). Lactate stimulates CA IX expression in normoxic cancer cells. Oncotarget.

[12] Lee DC (2015). Lactate-induced response to hypoxia. Cell.

[13] Hui S (2017). Glucose feeds the TCA cycle via circulating lactate. Nature.

[14] Faubert B (2017). Lactate metabolism in human lung tumors. Cell.

[15] de Saedeleer CJ (2012). Lactate activates HIF-1 in oxidative but not in Warburg-phenotype human tumor cells. Plos One.

[16] Ali MA, Konishi T (1998). Enhancement of hydoxyl radical generation in the Fenton reaction by alpha-hydroxy acid. Biochem. Mol. Biol. Int..

[17] Sonveaux P (2008). Targeting lactate-fueled respiration selectively kills hypoxic tumor cells in mice. J. Clin. Invest..

[18] Lanning NJ (2014). A mitochondrial RNAi screen defines cellular bioenergetic determinants and identifies an adenylate kinase as a key regulator of ATP levels. Cell Rep.

[19] Folmes CD, Dzeja PP, Nelson TJ, Terzic A (2012). Metabolic plasticity in stem cell homeostasis and differentiation. Cell Stem.

[20] Robinton DA, Daley GQ (2012). The promise of induced pluripotent stem cells in research and therapy. Nature.

[21] Rao MS, Malik N (2012). Assessing iPSC reprogramming methods for their suitability in translational medicine. J. Cell. Biochem..

[22] Yoshihara M, Hayashizaki Y, Murakawa Y (2017). Genomic instability of iPSCs: challenges towards their clinical applications. Stem Cell Rev. Reports.

[23] Takahashi K, Yamanaka S (2006). Induction of pluripotent stem cells from mouse embryonic and adult fibroblast cultures by defined factors. Cell.

[24] Takahashi K (2007). Induction of pluripotent stem cells from adult human fibroblasts by defined factors. Cell.

[25] Schmidt R, Plath K (2012). The roles of the reprogramming factors Oct4, Sox2 and Klf4 in resetting the somatic cell epigenome during induced pluripotent stem cell generation. Genome Biol..

[26] Yu J (2007). Induced pluripotent stem cell lines derived from human somatic cells. Science.

[27] Prieto J (2018). MYC induces a hybrid energetics program early in cell reprogramming. Stem Cell Reports.

[28] Zhang J, Nuebel E, Daley GQ, Koehler CM, Teitell MA (2012). Metabolic regulation in pluripotent stem cells during reprogramming and self-renewal. Cell Stem Cell.

[29] Spyrou J, Gardner DK, Harvey AJ (2019). Metabolomic and transcriptional analyses reveal atmospheric oxygen during human induced pluripotent stem cell generation impairs metabolic reprogramming. Stem Cells.

[30] Zhu S (2010). Reprogramming of human primary somatic cells by Oct4 and chemical compounds. Cell Stem Cell.

[31] Lone A, Harris RA, Singh O, Betts DH, Cumming RC (2018). p66Shc activation promotes increased oxidative phosphorylation and renders CNS cells more vulnerable to amyloid beta toxicity. Sci. Rep..

[32] Dranka BP (2013). Assessing bioenergetic function in response to oxidative stress by metabolic profiling. Free Radic. Biol. Med..

[33] Zhou G, Meng S, Li Y, Ghebremariam YT, Cooke JP (2016). Optimal ROS signaling is critical for nuclear reprogramming. Cell Rep.

[34] Hawkins KE (2016). NRF2 orchestrates the metabolic shift during induced pluripotent stem cell reprogramming. Cell Rep.

[35] Ying Z (2016). Transient activation of mitoflashes modulates Nanog at the early phase of somatic cell reprogramming. Cell Metab..

[36] Kida YS (2015). ERRs mediate a metabolic switch required for somatic cell reprogramming to pluripotency. Cell Stem Cell.

[37] Nishimura K, Fukuda A, Hisatake K (2019). Mechanisms of the metabolic shift during somatic cell reprogramming. Int. J. Mol. Sci.

[38] Prigione A (2014). HIF-1α modulates cell fate reprogramming through early glycolytic shift and upregulation of PDK1-3 and PKM2. Stem Cells.

[39] Akram M (2013). Mini-review on glycolysis and cancer. J. Cancer Educ..

[40] Yang W (2012). ERK1/2-dependent phosphorylation and nuclear translocation of PKM2 promotes the Warburg effect. Nat. Cell Biol..

[41] Corbet C, Feron O (2017). Cancer cell metabolism and mitochondria: nutrient plasticity for TCA cycle fueling. Biochim. Biophys. Acta - Rev. Cancer.

[42] Pires BRB (2014). The hypoxia-inducible factor-1α signaling pathway and its relation to cancer and immunology. Am. J. Immunol..

[43] Jung S (2008). Reactive oxygen species stabilize hypoxia-inducible factor-1 alpha protein and stimulate transcriptional activity via AMP-activated protein kinase in DU145 human prostate cancer cells. Carcinogenesis.

[44] Narita T (2009). Identification of a novel small molecular HIF-1α translation inhibitor. Clin. Cancer Res..

[45] Unternaehrer JJ (2014). The epithelial-mesenchymal transition factor SNAIL paradoxically enhances reprogramming. Stem Cell Reports.

[46] Esteban MA (2012). The mesenchymal-to-epithelial transition in somatic cell reprogramming. Curr. Opin. Genet. Dev.

[47] Roche TE, Hiromasa Y (2007). Pyruvate dehydrogenase kinase regulatory mechanisms and inhibition in treating diabetes, heart ischemia, and cancer. Cell. Mol. Life Sci..

[48] Zhang D (2019). Metabolic regulation of gene expression by histone lactylation. Nature.

[49] Zhou Y (2017). Mitochondrial spare respiratory capacity is negatively correlated with nuclear reprogramming efficiency. Stem Cells Dev..

[50] Ahn WS (2018). Glyceraldehyde 3-phosphate dehydrogenase modulates nonoxidative pentose phosphate pathway to provide anabolic precursors in hypoxic tumor cells. AIChE J..

[51] Jiang P, Du W, Wu M (2014). Regulation of the pentose phosphate pathway in cancer. Protein Cell.

[52] Birben E, Sahiner UM, Sackesen C, Erzurum S, Kalayci O (2012). Oxidative stress and antioxidant defense. World Allergy Organ. J.

[53] Holzerová E, Prokisch H (2015). Mitochondria: much ado about nothing? how dangerous is reactive oxygen species production?. Int. J. Biochem. Cell Biol..

[54] Sena LA, Chandel NS (2012). Review Physiological Roles of Mitochondrial Reactive Oxygen Species. Mol. Cell.

[55] Zorov DB, Juhaszova M, Sollott SJ (2014). Mitochondrial reactive oxygen species (ROS) and ROS-induced ROS release. Physiol. Rev..

[56] Rottenberg H, Hoek JB (2017). The path from mitochondrial ROS to aging runs through the mitochondrial permeability transition pore. Aging Cell.

[57] Zorov DB, Filburn CR, Klotz LO, Zweier JL, Sollott SJ (2000). Reactive oxygen species (ROS)-induced ROS release: a new phenomenon accompanying induction of the mitochondrial permeability transition in cardiac myocytes. J. Exp. Med..

[58] Kanti Das T, Wati MR, Fatima-Shad K (2014). Oxidative stress gated by Fenton and Haber Weiss reactions and its association with Alzheimer’s disease. Arch. Neurosci..

[59] Ying Z (2018). Short-term mitochondrial permeability transition pore opening modulates histone lysine methylation at the early phase of somatic cell reprogramming. Cell Metab..

[60] Ali MA, Yasui F, Matsugo S, Konishi T (2000). The lactate-dependent enhancement of hydroxyl radical generation by the Fenton reaction. Free Radic. Res..

[61] Ivan M (2002). Biochemical purification and pharmacological inhibition of a mammalian prolyl hydroxylase acting on hypoxia-inducible factor. PNAS.

[62] Choudhry H, Harris AL (2018). Advances in hypoxia-inducible factor biology. Cell Metab..

[63] Sonveaux P (2012). Targeting the lactate transporter MCT1 in endothelial cells inhibits lactate-induced HIF-1 activation and tumor angiogenesis. Plos One.

[64] Pagé EL, Chan DA, Giaccia AJ, Levine M, Richard DE (2008). Hypoxia-inducible Factor-1α stabilization in nonhypoxic conditions: role of oxidation and intracellular ascorbate depletion. Mol. Biol. Cell.

[65] Gerald D (2004). JunD reduces tumor angiogenesis by protecting cells from oxidative stress. Cell.

[66] Li X (2018). Upregulation of lactate-inducible snail protein suppresses oncogene-mediated senescence through p16 INK4a inactivation. J. Exp. Clin. Cancer Res..

[67] Melton C, Judson RL, Blelloch R (2010). Opposing microRNA families regulate self-renewal in mouse embryonic stem cells. Nature.

[68] Newman MA, Thomson JM, Hammond SM (2008). Lin-28 interaction with the Let-7 precursor loop mediates regulated microRNA processing. RNA.

[69] Zhang J (2016). LIN28 Regulates stem cell metabolism and conversion to primed pluripotency. Cell Stem Cell.

[70] Walenta S (2000). High lactate levels predict likelihood of metastases, tumor recurrence, and restricted patient survival in human cervical cancers. Cancer Res..

