# Supplementary material for: Lactate preconditioning promotes a HIF-1α-mediated metabolic shift from OXPHOS to glycolysis in normal human diploid fibroblasts
Source: Sci Rep. 2020 May 20;10:8388. doi: 10.1038/s41598-020-65193-9 (PMC7239882; doi:10.1038/s41598-020-65193-9)
Supplement: Supplementary file 1 — Supplementary information. [file 41598_2020_65193_MOESM1_ESM.pdf]

Lactate preconditioning promotes a HIF-1α-mediated metabolic shift from OXPHOS to glycolysis in normal human diploid fibroblasts

Alexandra M. Kozlov, Asad Lone, Dean H. Betts, and Robert C. Cumming

SUPPLEMENTARY INFORMATION

Figure Legends

Supplementary Figure S1. Defined metabolite treatment does not impact the transcript abundance of TCA cycle genes in human fibroblasts. BJ fibroblasts were cultured in defined metabolite media for 24 h prior to RNA isolation. qRT-PCR analysis using *ACTB* and *RPL37A* as housekeeping genes revealed that *ACLY, IDH1, OGDH, SDHB* and *MDH1* transcript abundance remained unchanged following defined metabolite treatment compared to control. The data presented represent N=3 ± s.e.m. One-way ANOVA and Dunnett's multiple comparisons test were used to determine significance with an alpha of 0.05. ns = no difference.

Supplementary Figure S2. Treatment with glucose following 20 h lactate exposure induces an increased ratio of phosphorylated PDH to total PDH in human fibroblast cells. To determine if lactate treatment promotes a sustained metabolic shift from OXPHOS to glycolysis, BJ cells were pre-treated in either glucose or lactate medium for 12, 16, 20 or 24 h prior to 48 h treatment with glucose media. Immunoblot analysis of the phosphorylation status of PDH was performed. Densitometric analysis of the ratio of ser_{232}-PDH to total PDH band intensities normalized to β-Actin, revealed that BJ cells cultured in lactate for 20 h prior to glucose exhibit a significantly increased ratio of phosphorylated PDH to total PDH compared to cells cultured only in glucose medium. The immunoblots presented are representative images for three independent experiments. Full length blots can be found in Supplementary Figure S4. The data presented represent N=3 ± s.e.m. Asterisks indicate significant difference ($p<0.05$ = *, $p<0.01$ = **) and ns = no difference tested by an Unpaired Two-tailed student's t-test.

Supplementary Figure S3. Treatment with KC7F2 reduces HIF-1α protein levels. BJ cells were cultured in DMEM under hypoxic conditions (1% O_2) for 12 h with and without the HIF-1α inhibitor KC7F2 to determine the effect of KC7F2 on HIF-1α protein levels. Immunoblot analysis of HIF-1α was performed. Densitometric analysis of HIF-1α band intensities normalized to β-Actin, revealed that culturing BJ cells in DMEM supplemented with 20 μM KC7F2 for 12 h was sufficient to reduce HIF-1α protein levels. The immunoblots presented are representative images from three independent experiments. Full length blots can be found in Supplementary Figure S4. The data presented represent N=3 ± s.e.m. Asterisks indicate significant difference ($p<0.01$= **) tested by an Unpaired Two-tailed student's t-test.

Supplementary Figure S4. Full length immunoblots. Red boxes surround the cropped portions of each immunoblot displayed in the manuscript figures and supplementary figures.

Supplementary Table S1. TaqMan™ Gene Expression Assays used for qRT-PCR analyses.

Supplementary Figure S1

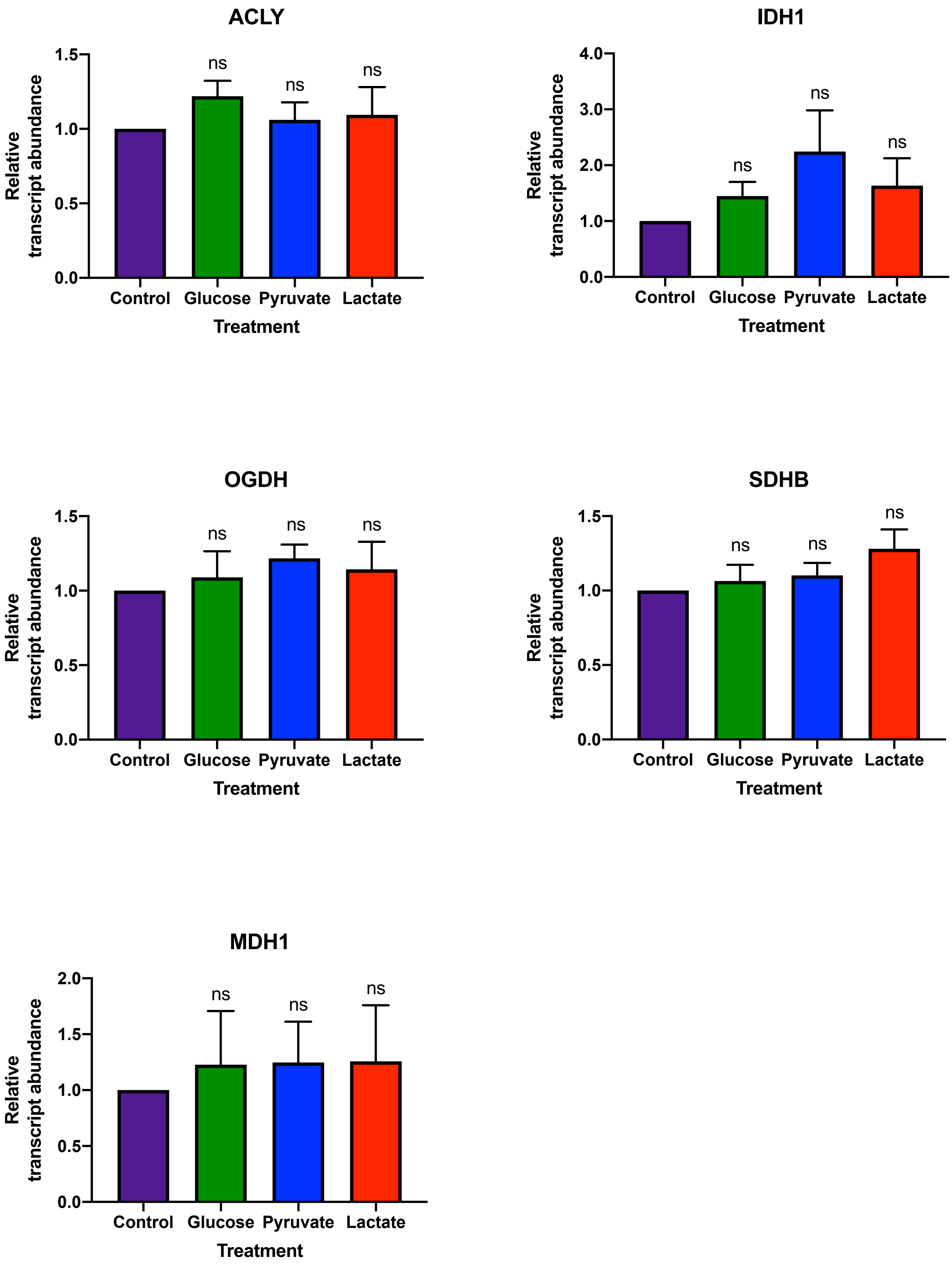

Supplementary Figure S2

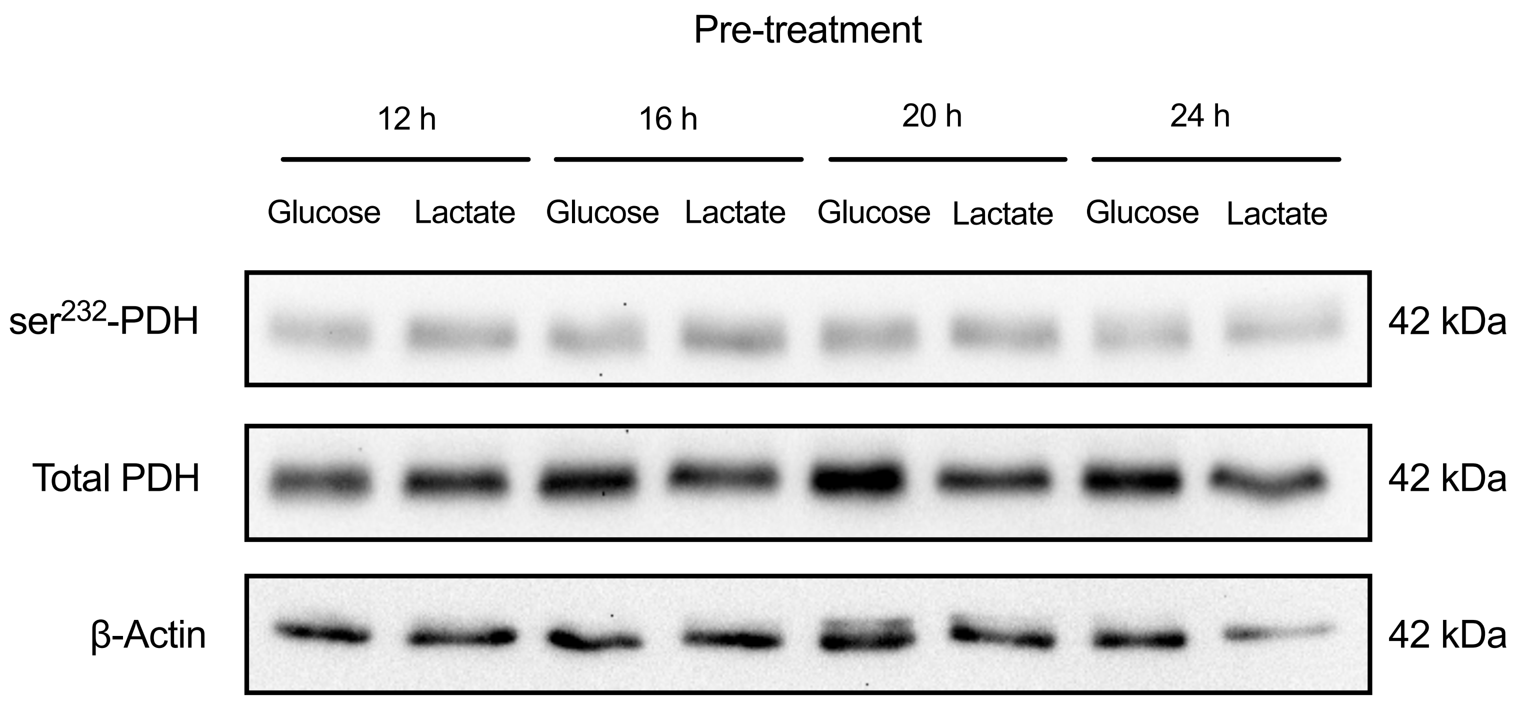

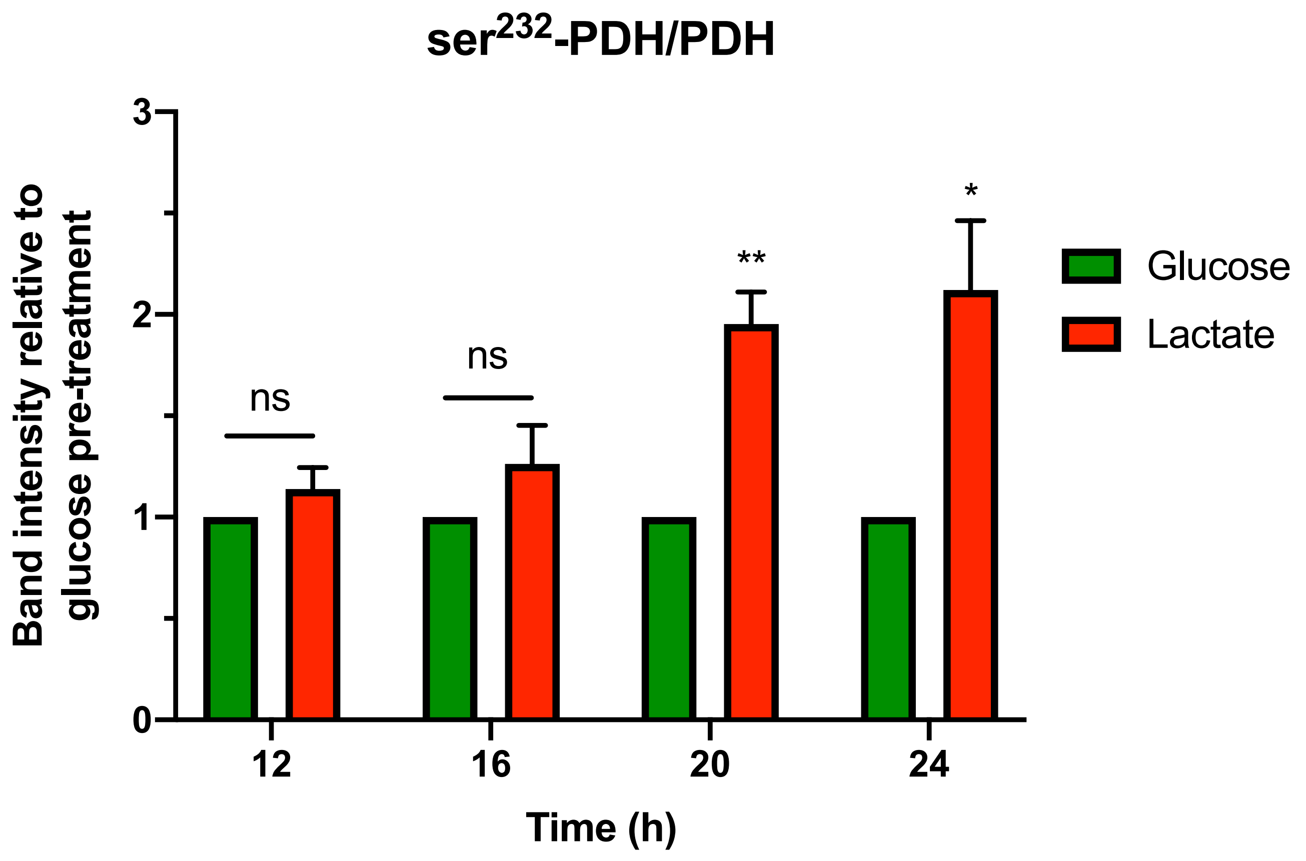

Supplementary Figure S3

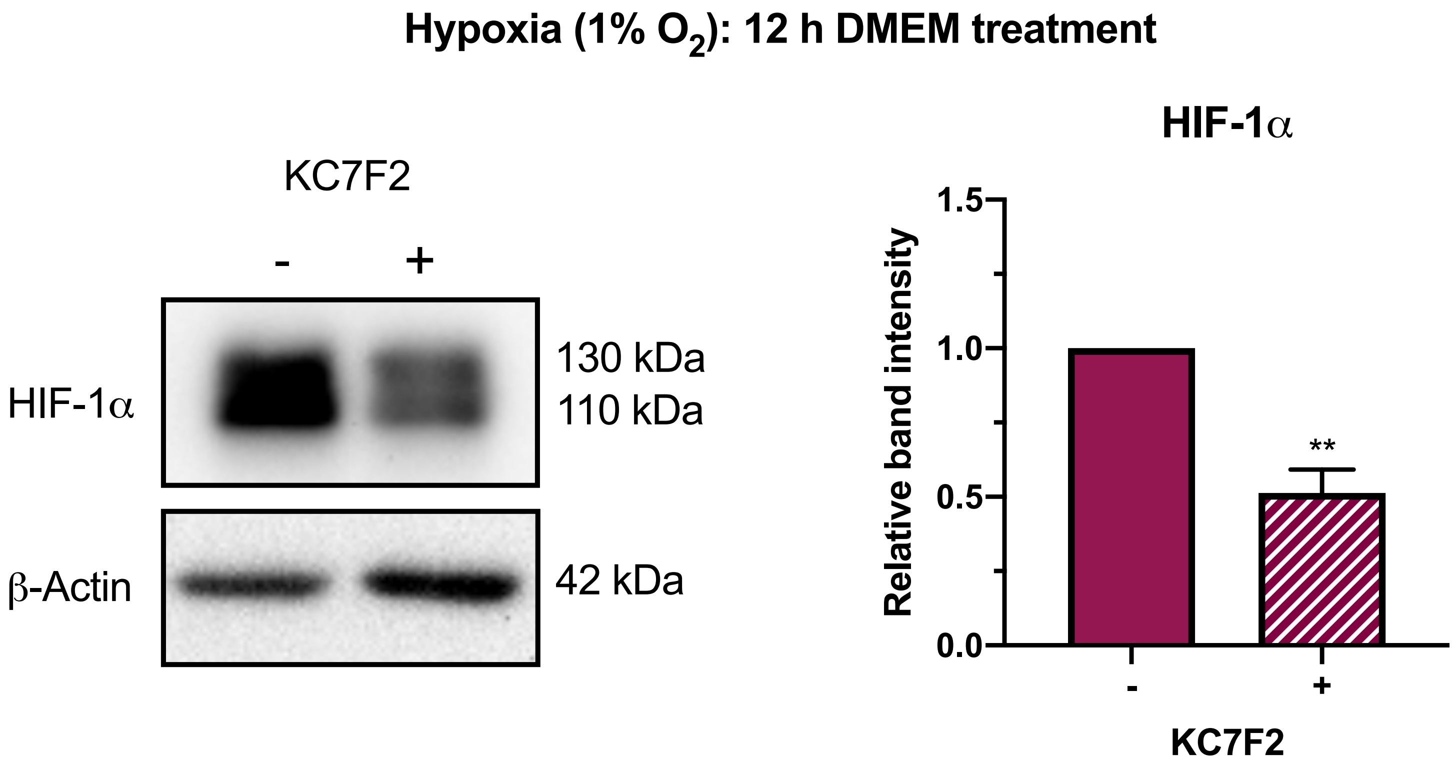

Supplementary Figure S4

Figure 1a

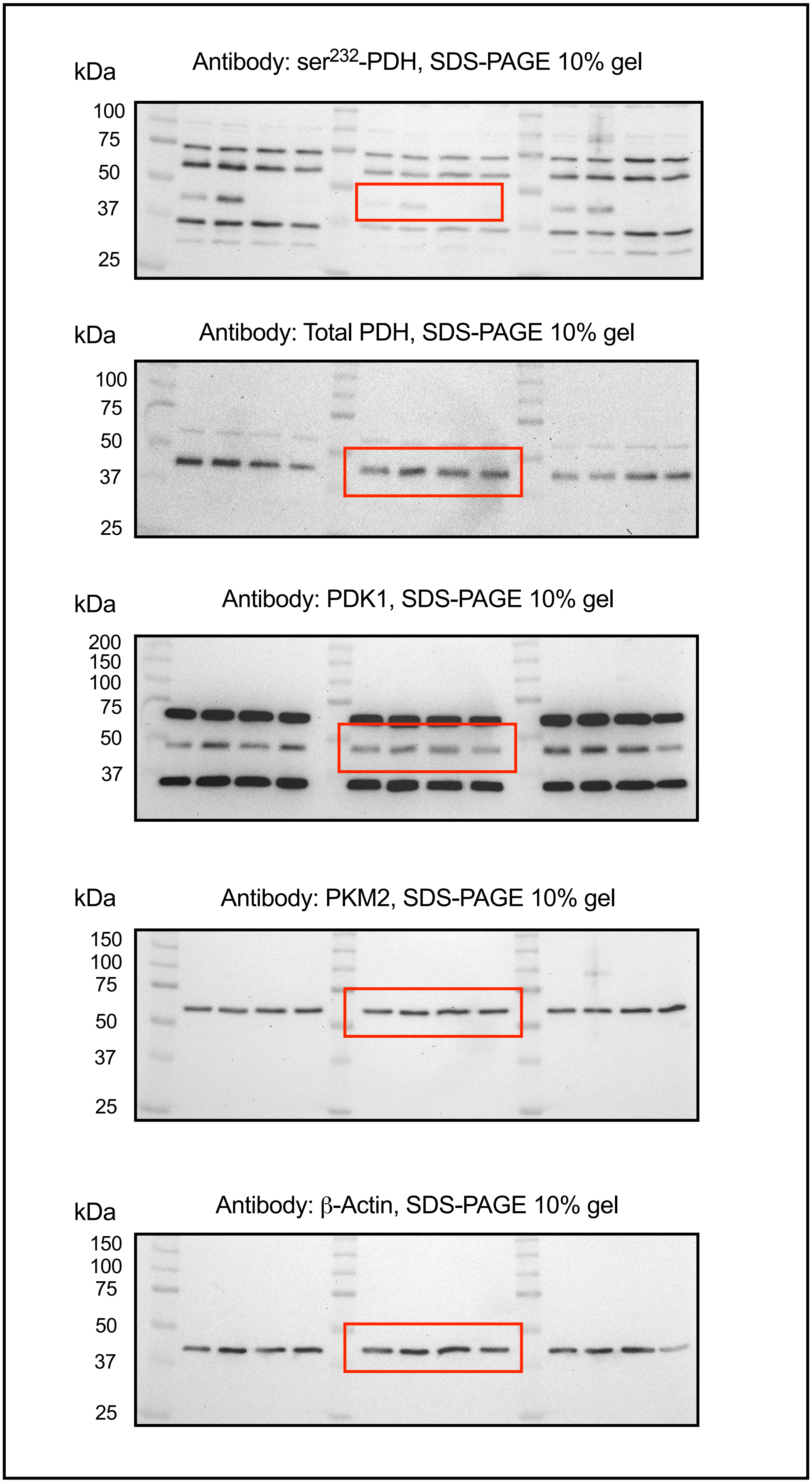

Supplementary Figure S4 continued

Figure 4a

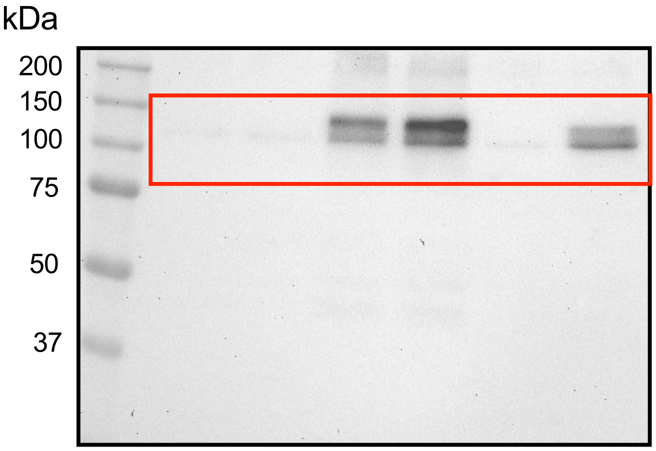

Antibody: HIF-1α, SDS-PAGE 10% gel

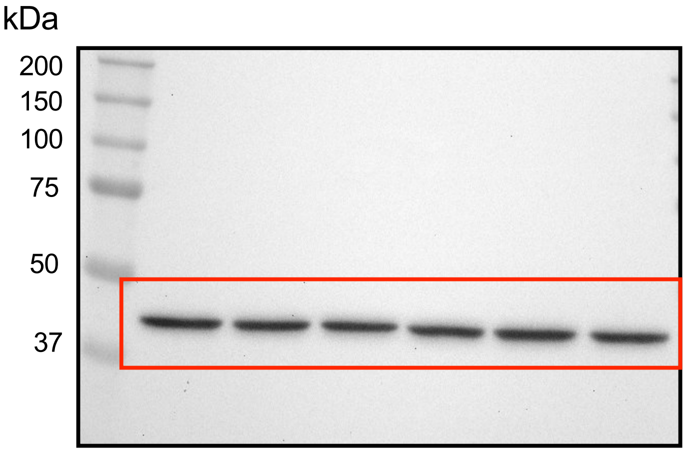

Antibody: β-Actin, SDS-PAGE 10% gel

Figure 4b (left), c (right)

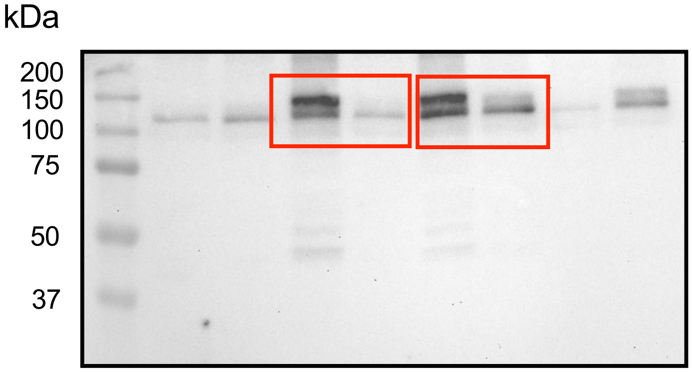

Antibody: HIF-1α, SDS-PAGE 10% gel

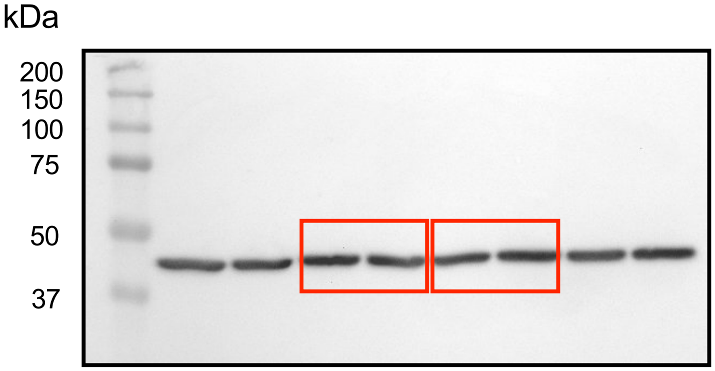

Antibody: β-Actin, SDS-PAGE 10% gel

Supplementary Figure S4 continued

Figure 5a & c

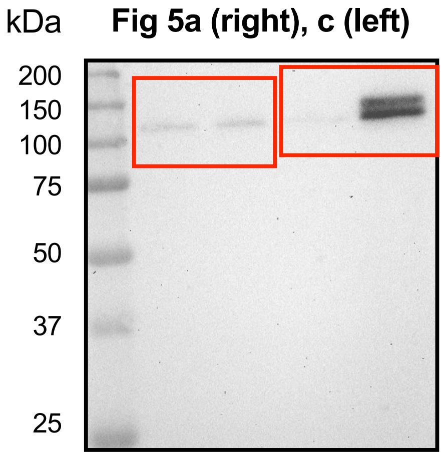

Antibody: HIF-1α,
SDS-PAGE 10% gel

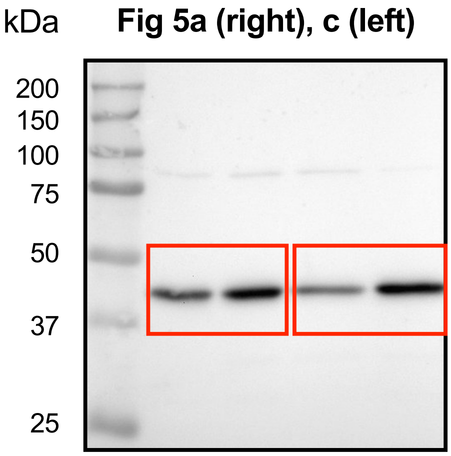

Antibody: PDK1,
SDS-PAGE 10% gel

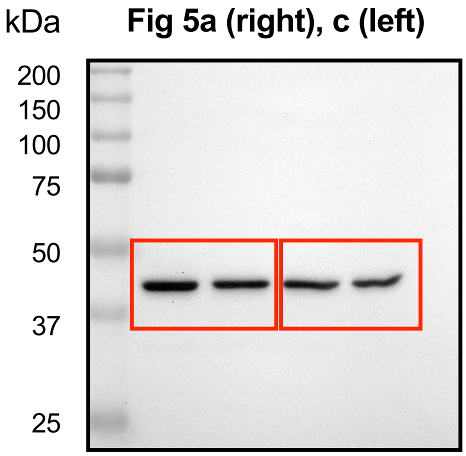

Antibody: β-Actin,
SDS-PAGE 10% gel

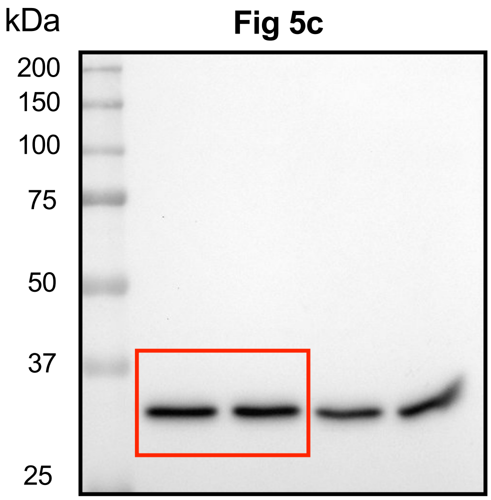

Antibody: LDHA,
SDS-PAGE 10% gel

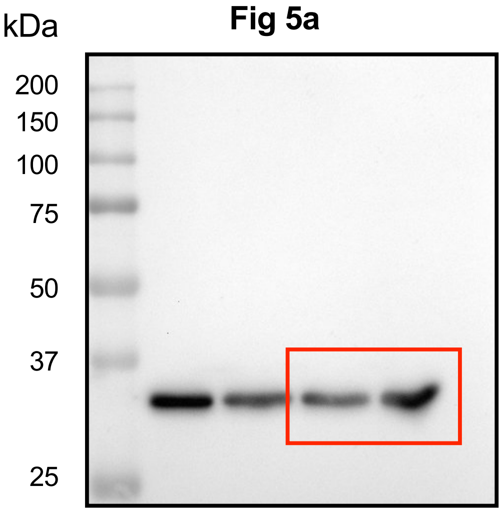

Antibody: LDHA,
SDS-PAGE 10% gel

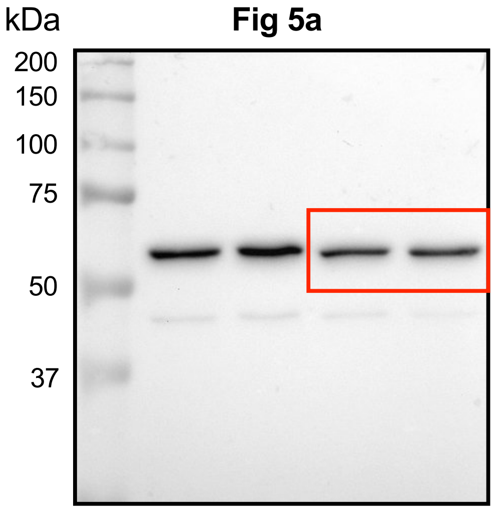

Antibody: PKM2,
SDS-PAGE 10% gel

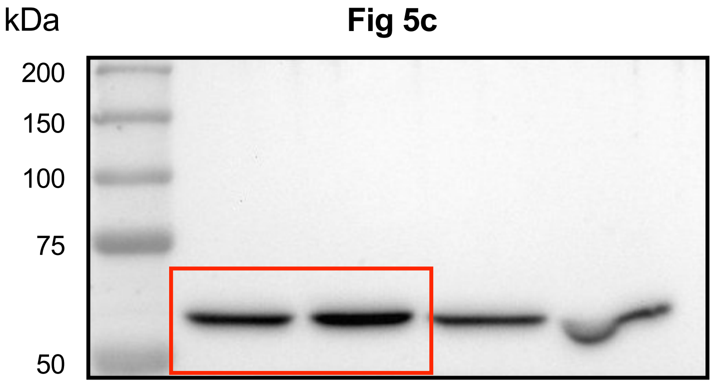

Antibody: PKM2, SDS-PAGE 10% gel

Supplementary Figure S4 continued

Figure 6

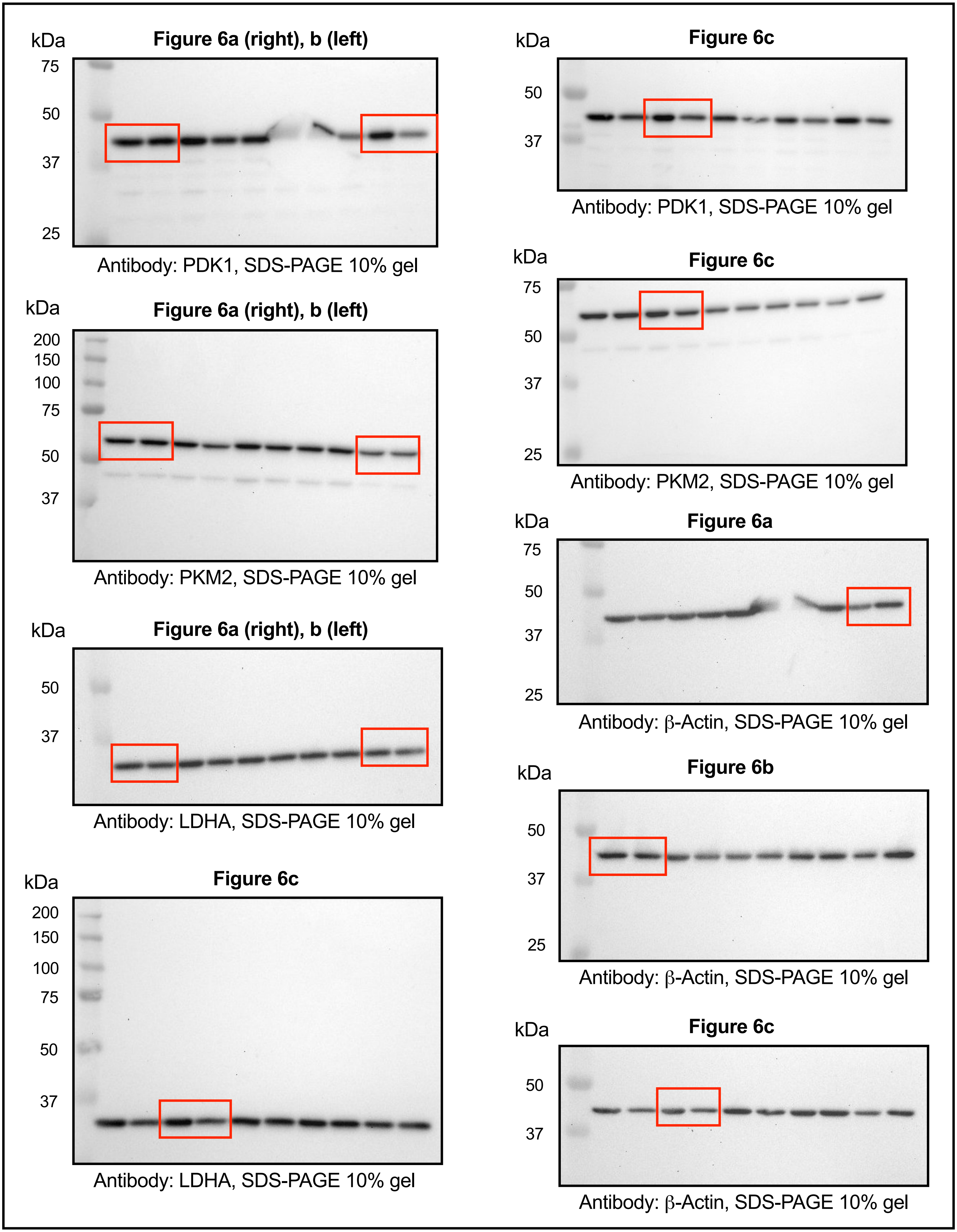

Supplementary Figure S4 continued

Supplementary Figure S2

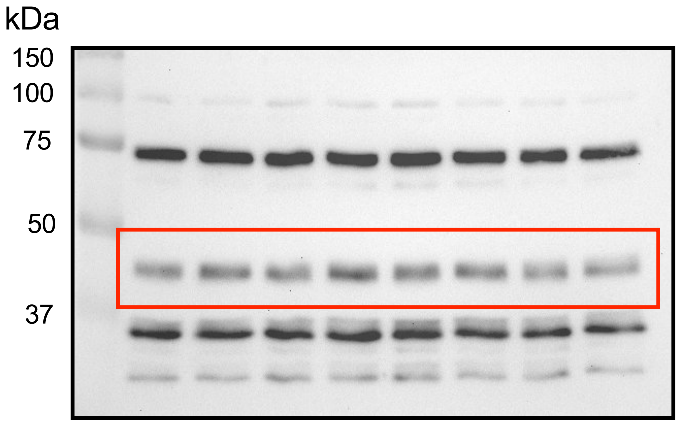

Antibody: ser^{232}-PDH, SDS-PAGE 10% gel

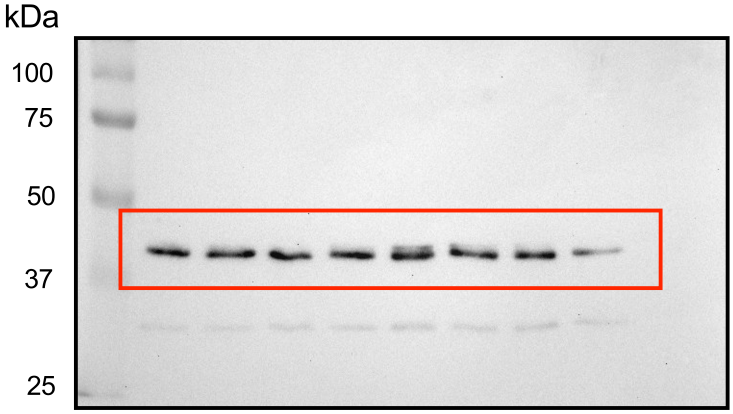

Antibody: β-Actin, SDS-PAGE 10% gel

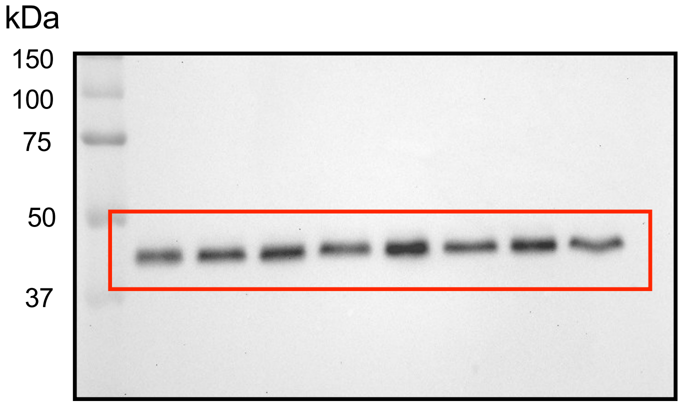

Antibody: Total PDH, SDS-PAGE 10% gel

Supplementary Figure S3

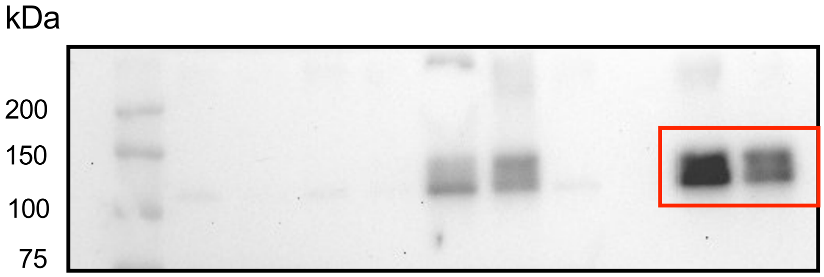

Antibody: HIF-1α, SDS-PAGE 10% gel

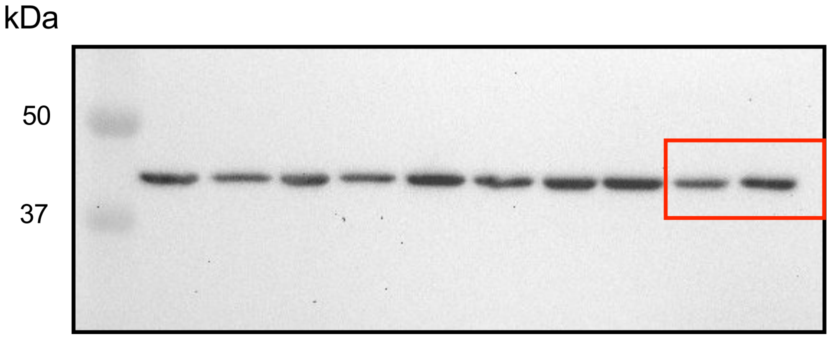

Antibody: β-Actin, SDS-PAGE 10% gel

Supplementary Table S1

Gene Symbol	Assay ID	Dye Label	Catalog Number
LDHA	Hs01378790_g1	FAM-MGB	4448892
PDK1	Hs01561847_m1	FAM-MGB	4448892
PKM	Hs00761782_s1	FAM-MGB	4448892
GAPDH	Hs02786624_g1	FAM-MGB	4453320
HK2	Hs00606086_m1	FAM-MGB	4453320
PGK1	Hs00943178_g1	FAM-MGB	4453320
ACLY	Hs00982738_m1	FAM-MGB	4453320
IDH1	Hs04966975_g1	FAM-MGB	4448892
OGDH	Hs01081865_m1	FAM-MGB	4448892
MDH1	Hs00936497_g1	FAM-MGB	4448892
SDHB	Hs00268117_m1	FAM-MGB	4448892
MYC	Hs00153408_m1	FAM-MGB	4453320
SNAI1	Hs00195591_m1	FAM-MGB	4453320
ESRRA	Hs00607062_gH	FAM-MGB	4448892
PPARGC1B	Hs00993805_m1	FAM-MGB	4448892
RPL37A	Hs01102345_m1	FAM-MGB	4331182
ACTB	Hs99999903_m1	FAM-MGB	4331182
HPRT1	Hs99999909_m1	FAM-MGB	4331182
